# Support Strategies and Interventions for eHealth Inclusion: Scoping Review

**DOI:** 10.2196/79760

**Published:** 2025-12-12

**Authors:** Wieke E Bouwes, Nynke D Scherpbier, Eveline Hage, Luc A R Tjalma, Esther I Metting

**Affiliations:** 1 Department of Primary and Long-Term Care, University Medical Center Groningen, University of Groningen, Post Code FA21, PO Box 196, Groningen, 9700 AD, The Netherlands, +31 652903891; 2 Data Science Center in Health (DASH), University Medical Center Groningen, University of Groningen, Groningen, The Netherlands; 3 Department of Innovation Management and Strategy, Faculty of Economics and Business, University of Groningen, Groningen, The Netherlands; 4 Department of Operations, Faculty of Economics and Business, University of Groningen, Groningen, The Netherlands

**Keywords:** eHealth, support strategy, support interventions, adoption, use, skills, attitudes, PRISMA

## Abstract

**Background:**

Policymakers increasingly promote eHealth as a way to improve health care efficiency. However, digitalization risks excluding individuals and groups who cannot fully engage with eHealth, for example, due to limited digital literacy or restricted access to resources. Targeted support, such as skills training, personalized guidance, or system-level initiatives, may help, but evidence on how such support is organized and on their outcomes for eHealth inclusion remains limited.

**Objective:**

This scoping review aimed to map proposed strategies to promote eHealth inclusion, identify concrete support interventions, and report evidence on their outcomes.

**Methods:**

This scoping review followed the PRISMA-ScR guidelines (Preferred Reporting Items for Systematic Reviews and Meta-Analyses extension for Scoping Reviews). Our search included PubMed, Scopus, Web of Science, and Embase for peer-reviewed studies published from 2014 onward, and the search was completed on January 29, 2024. We included empirical studies reporting on support to enhance eHealth inclusion. In total, 40 studies met the criteria: 19 examined support strategies and 21 evaluated targeted interventions. Strategies and interventions were categorized by actors at the microlevel (interpersonal, such as family members, friends, or peers), mesolevel (organizations, such as health care organizations or community organizations), and macrolevel (policy or system).

**Results:**

Support strategies and interventions addressed a range of eHealth types, including video consultations, mobile health applications, and patient portals. Strategy studies often emphasized interpersonal support from family, friends, or peers, whereas interventions more often involved health care providers. Intervention outcomes, as identified during analysis, were grouped into adoption, use, skills, and attitudes. Adoption-focused interventions led by health care organizations showed limited or not statistically demonstrated effectiveness. Interventions targeting use partly demonstrated positive effects, such as increased completion of video visits, whereas outcomes related to attitudes were mixed. Nearly all multiactor interventions—combining efforts across micro-, meso-, and macrolevels—effectively improved eHealth skills, including digital and eHealth literacy. Examples include programs linking health care providers with community organizations and initiatives pairing students with older adults, both of which improved these skills. Regional differences were also observed: health care providers played a dominant role in studies from the United States, community organizations were more prominent in African contexts, and multiactor approaches were common in European studies.

**Conclusions:**

Overall, interventions yielded mixed results, but multiactor collaborations frequently improved eHealth skills. These findings underscore the value of combining interpersonal, organizational, and policy-level efforts when designing support structures. For health care organizations, initiatives led solely by health care actors may suffice for promoting the use of specific applications (such as video consultations), but they seem insufficient for fostering broader eHealth literacy. Future research should address the sustainability and scalability of multiactor interventions and how health system contexts and cultural factors shape their outcomes.

## Introduction

An aging population and the rising burdens of noncommunicable diseases are increasing health care demand [1]. Global workforce shortages exacerbate resulting challenges for the health care system [1,2]. In response, policymakers and health care organizations increasingly advocate for the use of eHealth: technology to deliver and enhance health care services, incorporating a mindset to improve health care globally [3]. An example of eHealth is the use of personal health records (PHRs), which allow patients to access and review their medical history, laboratory results, and medication lists at any time [4]. PHRs also enable patients to share relevant information with different health care providers, which can support continuity of care across settings [5]. In emergencies, mobile PHRs provide timely access to critical information such as allergies or current medications, empowering patients to take a more active role in their own care [4].

Despite its potential, the benefits of eHealth are not equally distributed. Part of the population faces barriers to access or use eHealth, including low confidence, limited digital experience, low education, comorbidities, low socioeconomic status, older age, and being housebound [6,7]. These factors are closely linked to low eHealth literacy, defined as the ability to seek, find, understand, and apply health information from electronic sources to one’s health situation [8]. Evidence from a systematic review and meta-analysis demonstrates that eHealth literacy scores tend to decline when age increases, while higher educational attainment is associated with higher eHealth literacy. In contrast, living alone or lacking social support is linked to lower scores [9]. Low socioeconomic status and older age are also risk factors for specific chronic disease development and multimorbidity. As a result, the individuals most at risk of exclusion are also those who may benefit most from eHealth, for instance, through remote monitoring of chronic conditions [10,11]. This reflects the “inverse care law,” which states that those with the greatest need for care often have the least access to it [12].

The term “digital divide” has traditionally described a gap between those with and without access to eHealth [13]. However, the term is criticized for its binary perspective, which does not distinguish differences in people’s abilities to access eHealth across a spectrum [14]. Therefore, we use the term eHealth inclusion, derived from digital inclusion, which emphasizes having the ability to do what one wants or needs to do with eHealth [15].

Various fragmented support interventions, such as library courses, aim to enhance eHealth inclusion for those currently excluded [16]. However, there is a lack of research into their outcomes for eHealth inclusion and the role of collaboration with other support actors. This review aims to map existing support strategies and examine the outcomes of concrete support interventions that promote eHealth inclusion.

The research questions (RQs) are: (RQ1) What support strategies have been proposed to promote eHealth inclusion? (RQ2) How do concrete support interventions relate to these strategies, and what evidence exists regarding their effectiveness?

By combining the nonadoption, abandonment, scale-up, spread, and sustainability (NASSS) and population, intervention, and outcome (PIO) frameworks, we aimed to offer a comprehensive overview of support for eHealth inclusion across the micro-, meso-, and macrolevels. While the NASSS framework provides a holistic structure to analyze the context in which support is provided [17], the PIO framework enables a structured focus on the population, intervention, and outcomes reported in studies on support. The Methods section outlines how both frameworks were combined to guide data extraction and analysis. Overall, the aim of this review is to generate insights on eHealth support organizations and effects on eHealth inclusion that can guide policymakers, health care organizations, and other stakeholders in establishing support structures for accessible and inclusive eHealth.

## Methods

### Overview

This review is registered within the Open Science Framework (OSF) [18], and adheres to the PRISMA-ScR (Preferred Reporting Items for Systematic reviews and Meta-Analyses extension for Scoping Reviews) [19]. Since we aimed to map all existing evidence on eHealth support regardless of methodological quality and risk of bias, critical appraisal—an optional component of the PRISMA-ScR—was not conducted. Our review follows the step guide of Arksey and O’Malley [20], comprising five stages: (1) identifying the research question, (2) identifying relevant studies, (3) performing the study selection, (4) charting the data, and (5) summarizing and reporting the results [20].

We have chosen to conduct a scoping review to map the diversity of research available on eHealth support. Following Arksey and O’Malley’s framework [20], scoping reviews can be performed for several reasons. Our review addresses two: (1) providing an overview of the extent, range, and nature of research activity on a broad topic, and (2) identifying research gaps. This approach is particularly suited to eHealth support, as the field is broad and initiatives are scattered across domains.

Throughout the review, two types of studies are considered:

1. “Strategy studies” propose ways to organize and provide support, via noninterventionist approaches, looking at the target group’s existing environmental influences on eHealth inclusion. While mostly exploratory, some studies use quantitative methods, such as surveys.
2. “Intervention studies” evaluate targeted support interventions. Specifically, these studies report outcomes related to eHealth inclusion, either descriptively or through statistical testing.

### Eligibility Criteria

Eligible studies were empirical, written in English, published in peer-reviewed journals, from 2014 onwards, meeting the population, intervention, and outcome (PIO) criteria. Studies included those targeting (potential) patients (population) receiving help or support (intervention), with reported outcomes related to eHealth inclusion (outcome). We defined eHealth inclusion as accessibility, (digital or eHealth) literacy, and overall inclusion. In the included studies, related outcomes such as adoption, frequency of use, user satisfaction, and digital skills were also reported. These were considered part of the broader conceptualization of eHealth inclusion and were included in our outcome categorization.

Our review focused exclusively on peer-reviewed studies, as our research questions concerned both proposed strategies (RQ1) and evidence of the effectiveness of support (RQ2). Peer-reviewed publications provide a transparent and reproducible source of such evidence. Gray literature was excluded to maintain methodological transparency and reproducibility.

### Search Strategy and Selection Criteria

After consultation with a librarian, search terms were developed based on the PIO framework, resulting in 3 search strings. The population terms, linked using the OR operator, included: “patient*,” “people,” “client*,” “care consumer*,” “care receiver*,” and “older adult*.” The intervention string consisted of terms related to support, combined with “intervention*,” “network,” and “system.” The outcome string included synonyms for “telemedicine,” “eHealth,” and “mobile health,” as well as terms related to “health literacy,” “inclusion,” and “accessibility.” These strings were combined using an “AND” operator. The complete search strategy can be found in Multimedia Appendix 1.

### Data Extraction

**Figure 1.**
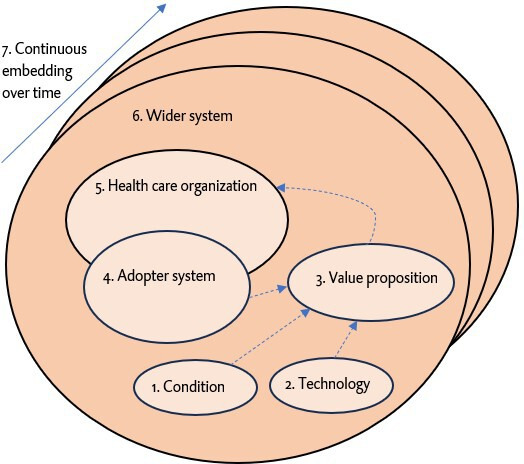
Greenhalgh and colleagues’ [17] nonadoption, abandonment, scale-up, spread, and sustainability (NASSS) framework, applied in this scoping review (2014‐2024) on support strategies and interventions for patients’ eHealth inclusion. The framework was used in combination with the population, intervention, and outcome (PIO) framework to categorize intervention and strategy study items.

Data were extracted from the included records based on a personalized configuration of the NASSS and PIO framework. Figure 1 shows the NASSS framework [17]. Table 1 shows how the NASSS dimensions were combined and interpreted in this review. In addition, Multimedia Appendix 2 shows the items for which data were extracted. For strategy studies, data were extracted on the population and intervention only, as these studies typically proposed forms of support without reporting outcomes. For intervention studies, data were extracted based on the full PIO criteria, since these studies reported outcomes related to eHealth inclusion, either descriptively or through statistical testing. We also classified countries of the studies by region according to the United Nations geoscheme. To gain insight into health system characteristics, we drew on the Commonwealth Fund’s International Profiles of Health Care Systems of 2015 and 2020 [21,22]. Multicountry studies were counted once overall. In Multimedia Appendix 3, we present the context of the different countries within the study row. Data extraction was primarily performed by WEB, in consultation with authors EH, EIM, and NDS.

**Table 1.** Operationalization of concepts to categorize eHealth support interventions and strategies to promote patients’ eHealth inclusion, based on the population, intervention, and outcome (PIO) and nonadoption, abandonment, scale-up, spread, and sustainability (NASSS) frameworks, applied in this scoping review (2014‐2024) of studies involving diverse patient populations.

PIO^a^ and NASSS^b^ concepts		Operationalization
Population		
	Condition (population): Illness at stake.	Target group (population): The population targeted: defined by age, illness, or other characteristics.
	The technology (population): (2A) Material features, (2B) knowledge generated by the technology, (2C) knowledge required for use, and (2D) issues for sustaining the technology.	Type of eHealth (population): The specific form of technology at stake (eg, video visits, patient portals, etc).
	The adopter system (population): Adoption of the technology by patients and lay caregivers.	Users of the eHealth support (population)
Intervention		
	The adopter system (population and intervention): Adoption of the technology by patients and lay caregivers.	Co-users (e.g., proxy-users) Support actors (intervention): Those providing the support.
	The organization (intervention): Readiness and capacity of the organization, including adoption decisions and budget allocation.	Initiating organization (intervention): The organization where the support was initiated (eg, hospital and health center). Project (intervention): The initiated project. Project content (intervention): Available information on modules, steps, and content of the project.
	Value proposition: For whom the technology generates value, including benefits for patients or intended users.	Intended value (intervention): The intended value of the study and support.
	The wider context (intervention): Institutional and sociocultural context (including health policy), as per Greenhalgh et al [17].	External stakeholders (intervention): Contributing to the sociocultural spreading of the intervention, or wider institutional influences. Legislative context (intervention): Which policies are at stake in the wider context of the intervention? Temporal dynamics (intervention): This was freely added to describe the influence of temporal dynamics, such as COVID-19, as this is often found to be a large motivator to initiate a support intervention.
Outcome		
	Value proposition: For whom the technology generates value, including benefits for patients or intended users.	Realized value (outcome): The actual realized value or eHealth inclusion in the target group.
	Interaction between domains over time (outcome): Organizational resilience and feasibility in the medium to long term.	Future intentions (outcome): Future goals and (expected) feasibility over time.

a PIO: population, intervention, and outcome.

b NASSS: nonadoption, abandonment, scale-up, spread, and sustainability.

### Classification of Actors Involved in Interventions and Proposed in Strategy Studies

While the NASSS framework does not explicitly categorize support in the context of eHealth, its dimensions do address micro- (individual adoption), meso- (organizational assimilation), and macrolevel challenges (policy and regulatory environment) [17]. These levels are adopted to differentiate actors across levels for providing eHealth support.

## Results

### Search Results and Study Selection

**Figure 2.**
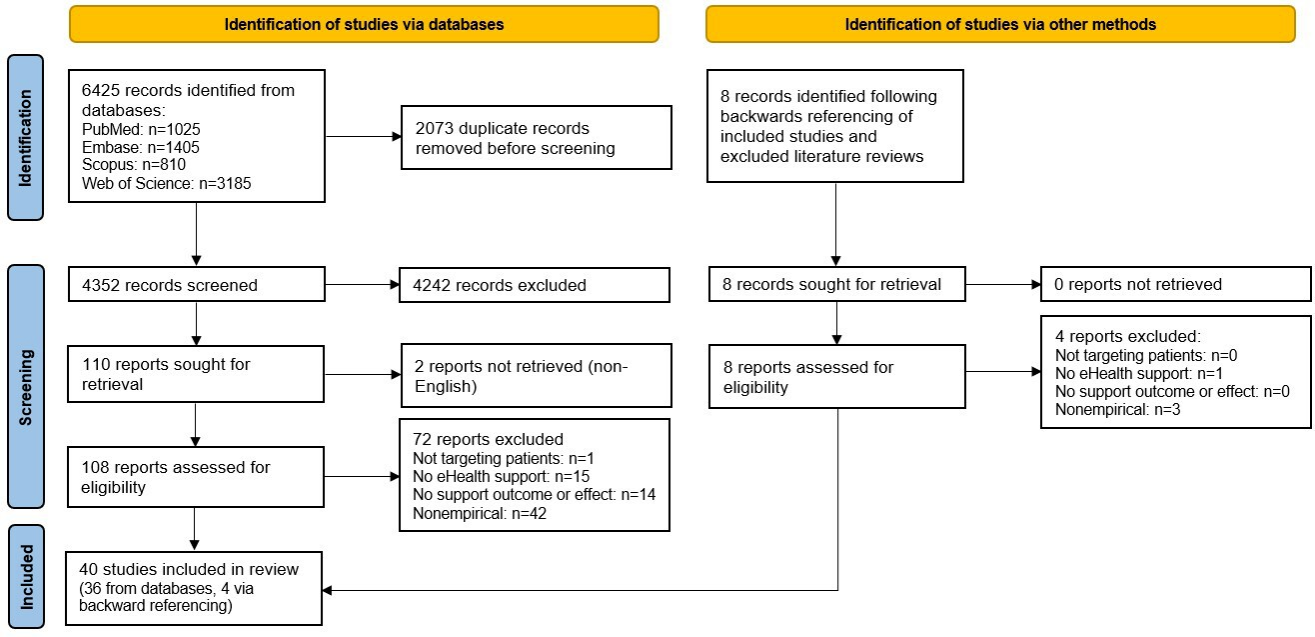
PRISMA (Preferred Reporting Items for Systematic Reviews and Meta-Analyses) flow diagram.

The PRISMA-ScR flowchart in Figure 2 illustrates the screening process. Deduplication was performed in EndNote (Clarivate) following the Bramer method [23] to remove duplicates. After deduplication, 4352 articles remained for title and abstract screening. Authors WEB and LART used Rayyan.ai, a web-based and mobile app developed to facilitate the screening process for reviews, to independently screen all titles and abstracts for inclusion based on the predefined criteria. Rayyan enables blinded screening by allowing reviewers to hide each other’s decisions during the screening process and allows reviewers to define and apply their own exclusion criteria [24].

Before screening, authors performed a pilot test on 20 random articles, yielding a 75% (15/20) agreement with a Cohen kappa of 0.42, indicating moderate agreement [25]. After discussing and refining the inclusion criteria, the authors independently screened all titles and abstracts, resulting in discrepancies for 28 cases, yielding a 99.4% (4324/4352 articles) agreement and a Cohen kappa of 0.99. The 28 discrepancies were resolved collaboratively, and 2 non-English articles were excluded, leaving 108 articles for full-text screening.

For the full-text articles, the authors again assessed agreement on a sample of 20 random articles, for which full consensus was achieved. After screening all 108 articles, 11 conflicts were found. A total of 10 of these were resolved by authors LART and WEB through discussion, and EIM made the final decision on the remaining articles. After this process, 36 articles remained, and backward reference checking resulted in 40 included studies [26,65].

### Classification of Included Studies

In Table 2, the included studies are categorized by population, intervention, and outcome. The second column summarizes the populations and interventions of the strategy studies, while the third column summarizes the populations, interventions, and outcomes of the intervention studies. Detailed descriptions of both study types are provided in MultimediaAppendices 4  5 [26-65].

**Table 2.** Population, intervention, and outcome (PIO)-based categorization of strategy studies (n=19) and intervention studies (n=21) included in this scoping review (2014‐2024), considering a range of patient populations, eHealth types, support actors, and eHealth inclusion outcomes.

PIO^a^ data	Strategy studies (n=19) [26-44], n (%)	Intervention studies (n=21) [45-65], n (%)
Population		
Target population		
Older adults or patients	10 (53) [27,29,31,32,34,36,37,41,43,44]	8 (38) [47,49,52,55-57,63,64]
All adults or patients (aged 18 years and older)	2 (11) [30,38]	6 (29) [45,46,50,51,53,60]
People of all ages	2 (11) [39,40]	2 (10) [54,61]
Patients with chronic diseases	7 (37) [26,33-35,41-43]	3 (14) [58,62,65]
Patient representatives	1 (5) [28]	0 (0)
Individuals with mental illnesses	0 (0)	2 (10) [45,48]
Community stakeholders	0 (0)	1 (5) [50]
Maternal health care clients	0 (0)	1 (5) [59]
eHealth type		
Broad or mixed eHealth	11 (58) [26,29,30,32,33,35-37,39,40,43]	6 (29) [47,50,56,57,64,65]
Video visits	3 (16) [27,28,38]	6 (29) [46,49,51,52,54,60]
Internet for health purposes	1 (5) [31]	1 (5) [55]
Mobile health applications	1 (5) [34]	5 (24) [45,48,53,59,63]
Patient portals	1 (5) [38]	3 (14) [58,61,62]
Internet for cardiovascular self-management	1 (5) [41]	0 (0)
eHealth for chronic disease management	1 (5) [42]	0 (0)
Intervention		
Interpersonal	11 (58) [26-32,34,35,37,43]	3 (14) [53,59,63]
Technology supplier: hardware	2 (11) [31,33]	3 (14) [54,57,59]
Technology supplier: software	2 (11) [28,31]	5 (24) [45,47,48,58,64]
Health care	7 (37) [29,30,33,38,40-42]	15 (71) [45,46,48-54,57,58,60-62,65]
Educational: commercial	2 (11) [27,32]	1 (5) [57]
Educational: public	0 (0)^b^	4 (19) [47,50,52,56]
Community	7 (37) [26-28,31,32,36,37]	8 (38) [45,46,48,50,55,57,59,63]
Policy intervention	5 (26) [26,33,37,39,44]	3 (14) [45,55,57]
Outcome (only for intervention studies)		
Amount of portal enrollment activations	N/A^c^	2 (10) [61,62]
Portal log-in (yes or no)	N/A	1 (5) [58]
Frequency of technology use	N/A	1 (5) [50]
Video visit completion	N/A	4 (19) [49,51,60,65]
Digital literacy	N/A	3 (14) [45,48,53]
eHealth literacy	N/A	2 (10) [47,56]
Telehealth competency	N/A	1 (5) [64]
Confidence in using technology	N/A	1 (5) [63]
Cognitive load reduction	N/A	1 (5) [46]
Comfort using technology	N/A	1 (5) [52]
Qualitative attitudes	N/A	4 (19) [54,55,57,59]

a PIO: population, intervention, and outcome.

b Not proposed or included in support.

c N/A: not applicable.

### Findings Part I: Strategy Studies

#### Populations Targeted (Population)

The strategy studies targeted diverse populations, categorized by age, disease, or both. A total of 9 studies directly and 1 indirectly (via their primary care physician) [27], focused on older adults and older patients [27,29,31,32,34,36,37,41,43,44]. The specified age cutoffs for these populations were: ages 45 years and older [36], 50 years and older [32], 55 years and older [29], 60 years and older [31,43,44], and 65 years and older [27,34,37,41]. Other age-focused populations included adults, with 1 study including adults ages 21 years and older [30], and another including patients ages 18 years and older [38]. A total of 2 studies included people of all ages [39,40].

A total of 8 studies focused on disease-specific populations, which were, for instance, patients with chronic conditions [33,42,43], ranging from adults aged 18 years and older and their primary care providers [33] to patients with chronic diseases together with their care managers [42]. Other target populations included the further specified chronic diseases: dementia [26], cardiovascular risk [41], and diabetes [34,35]. Furthermore, 1 study included patient representatives as their target group [28].

Moreover, 7 out of 19 studies focused on a specified type of eHealth, including video visits [27,28], internet use for health purposes [31], mobile health applications [34], both patient portals and video visits [38], an online platform for cardiovascular self-management [41], and eHealth for chronic disease management [42]. The remaining 12 studies did not specify types and rather addressed the broad category of eHealth [26,29,30,32,33,35-37,39,40,43].

#### Aims and Focus Areas

Strategy studies had diverse aims (see Multimedia Appendix 4 [26-44] for the intended value column), including to gain insights into telehealth use and needs among participating patients [38] and to explore which factors influence initial and sustained platform engagement [41]. Despite this variation in aims, all studies included strategic components to arrange support provision.

#### Proposed Strategies and Support Actors (Intervention)

Table 2 outlines the actors proposed to provide support throughout strategy studies. Table 3 shows how actors proposed in strategy studies could be classified into micro- (interpersonal), meso- (technology supplier, health care organization, educational organization, and community organization), and macrolevel (policy level) support. Technology suppliers were specified to provide hardware (loaning or providing devices and support) and software (online programs).

**Table 3.** Support actors proposed in strategy studies (n=19) included in this scoping review (2014‐2024) on support strategies and interventions for patients’ eHealth inclusion. The table categorizes proposed actors across micro-, meso-, and macrolevel (interpersonal, technology supplier—specified as hardware or software, health care, educational, community, and policy) as described in the included studies.

Study	Interpersonal	Technology supplier		Health care organization	Educational organization	Community organization	Policy level
Hardware	Software
Arighi et al [26]	✓					✓	✓
Chen et al [27]	✓				✓	✓	
Curran et al [28]	✓		✓			✓	
Han et al [29]	✓			✓			
Hayat et al [30]	✓			✓			
Hodge et al [31]	✓	✓	✓			✓	
Jokisch et al [32]	✓				✓	✓	
Khairat et al [33]		✓		✓			✓
Kim et al [34]	✓						
Lee et al [35]	✓						
Lin et al [36]						✓	
Marston et al [37]	✓					✓	✓
Pack et al [38]				✓			
Radovanovic et al [39]							✓
Shahid et al [40]				✓			
van Middelaar et al [41]				✓			
Williams et al [42]				✓			
Wu et al [43]	✓						
Zhao et al [44]							✓
Total, n (%)	11 (58)	2 (11)	2 (11)	7 (37)	2 (11)	7 (37)	5 (26)

#### Interpersonal Support (Micro) Most Frequently Proposed

A total of 11 studies proposed to involve interpersonal contacts in support provision, either in the form of social support, or specified to family or peer support [26-32,34,35,37,43].

##### Social Support

In total, 5 studies proposed social support. One of these highlighted social connections is crucial for self-learning, especially for those potential users with little eHealth experience [31]. Adding to this advice, other authors proposed to incorporate synergistic effects after finding an indirect positive relation of social support on the relationship between eHealth literacy and self-management among diabetes patients [35]. From a similar basis, another survey study proposed social support after finding a positive association between social support and self-management, mediated by eHealth literacy [43]. In addition, a study on design aesthetics unveiled that social support may enhance mobile health care use in diabetes patients [34]. Other authors discovered that while social support improved perceived ease of eHealth use in their sample, personal connections decline with age, stressing an additional need for formal support systems [29].

##### Family Support

More specifically, studies proposed family support based on different findings. For instance, a study splitting dementia participants into those able and those unable to complete a video visit independently discovered that the participants able to complete video visits were often assisted by a younger caregiver, such as children or grandchildren [26]. Other authors found that older adults seeking family support had higher intentions to use eHealth, though further research was recommended on its link to actual use [32]. Another study unveiled that physicians valued family involvement in video visit support alongside education and patient readiness assessments [27].

##### Peer Support

While 1 study did not find a significant effect of peer support on eHealth use [32], other studies did propose a role for peers. A phenomenological study observed that people in similar situations can educate others on eHealth, and peer success stories encourage adoption [28]. Similarly, researchers found that ties with similar others can promote useful information exchange, resulting in attitudes in favor of eHealth use. This study also indicated these relations could compensate for low eHealth literacy [30]. A study among participants from both rural and urban areas recommended peer-to-peer learning as an important way for older adults to develop confidence in using technology [37]. In contrast, the study that did not find such an effect was conducted among highly educated individuals at technology-related events, which the authors suggested may have biased the results [32].

### Organizational (Mesolevel Support)

Next to microsupport, studies also proposed to include support actors at the mesolevel, by focusing on various actor types described in this section.

#### Support of Technology Suppliers

Findings from 3 studies indicated a supporting role of technology suppliers [28,31,33]. A total of 2 of these focused on software delivery [31,33]. The first focused on older residents in a rural area and advised providers to ensure accessibility of support information as well as to deliver tailored training programs [31]. The second study proposed a web-based training as one of several formats to accommodate diverse learning styles [28]. The third study proposes hardware support of technology suppliers in providing easy-to-use devices and a helping kit for their target group at-home measurements [33].

#### Support by Health Care Organizations

Health care support was proposed in 7 studies [29,30,33,38,40-42]. Examples include home-based preparatory support [40], in-home testing and nurse evaluations when implementing remote monitoring devices [33], and the use of a platform facilitated by a patient’s known cardiovascular disease coach [41]. More specifically, a study distinguished users from nonusers and found that the majority of users reported having received assistance from health care actors. In this same study, 33.4% of nonusers desired in-home support from health care actors, leading the authors to propose a role for health care [38]. Another study, which proposed both a strategy and reported intervention outcomes, identified challenges related to smartphone use and connectivity in transitioning to eHealth. The authors proposed and piloted support interventions within health care organizations, which are briefly reported in the intervention part of the results [42]. However, as mentioned, the study is not included in the intervention count (n=21), since the main focus was to identify factors influencing implementation.

#### Support by Educational Organizations

A total of 2 studies proposed educational support, for instance, emphasizing nonformal education as valuable for those with lower education levels, suggesting voluntary programs could foster eHealth use when formal support sessions are inaccessible [32]. The other study proposed volunteering of medical students as a useful way to get patients to work on their eHealth skills [27].

#### Support by Community Organizations

A total of 7 studies suggested a role for community organizations [26-28,31,32,36,37]. One study proposed including community organizations in social safety nets to simulate intergenerational support [26]. A suggestion by another study involved engaging community organizations and senior groups to educate and encourage the use of eHealth and to support community-based knowledge sharing [28]. Other authors found that those seeking health information through library activities had higher eHealth literacy [36]. Specifically, those having more than 9 years of education, having a higher income, from an urban setting, and from nonfarm employment reported significantly better eHealth literacy scores than those who lack these characteristics. The study proposed opportunities for libraries to reach out to those lacking these characteristics [36]. Despite the proposed advantages of library use, challenges remain. While another study of a mostly highly educated sample found formal support programs (including from libraries) could positively influence eHealth literacy, the authors eventually advised nonformal education within interpersonal communities may better address the needs of more vulnerable groups, who may be less suited to fixed formal programs [32].

#### Policy Interventions (Marco-level Support)

The authors of one paper argued for establishing policy-led interventions to overcome inequalities in access to social support and solidify social safety nets for those needing support [26]. Other authors argued that even though interpersonal support is often built on trust, entirely relying on these actors was not perceived as beneficial, due to the time restrictions of the family. Therefore, authors stressed the need for governments to implement policies for older adults, allowing them to practice using eHealth [44]. Other authors argue policymakers should address digital literacy gaps by ensuring fair eHealth reimbursement for providers and helping to streamline technical support [33].

### Advice for the Design and Content of Support Provision

On top of identifying actors to provide eHealth support, broader recommendations exist, such as the stressed need for in-person support to overcome barriers for eHealth inclusion [38,39]. Also proposed are allowing blended models of care, where eHealth and physical care are combined to accommodate diverse patient needs [29,34,39,40]. Given differences in patient preferences and learning styles, studies emphasize the importance of providing materials in personalized, appropriate formats ranging from printed information booklets to spoken tutorials, and in-person at-home or clinic-based visits [29,34,39,40]. In the “Findings Part II: Intervention Studies” section, we report findings from intervention studies, which examined the outcomes of specific support strategies.

### Findings Part II: Intervention Studies

This section reports findings from intervention studies, for which we examined reported outcomes related to eHealth inclusion.

#### Populations Targeted (Population)

The specified target populations in the intervention studies varied from disease-focused to age-related and other populations. Disease-focused populations were cancer patients [65], diabetes patients [62], and adults with mental illnesses [45,48].

Older adults were defined using different age cut-offs: ages 50 years and older [47], 55 years and older [63], 60 years and older [57,64], and 65 years and older [49,52,55-57]. Other populations targeted adults [45,46,50,51,53,60], and people of all ages [54,61], including, for instance, maternal health care clients [59], primary care and geriatric patients [60], vulnerable patient groups [46], and patients with limited digital literacy [50]. Also included were community stakeholders [50].

Of the 21 studies evaluating support interventions, 6 studied broad or mixed eHealth [47,50,56,57,64,65], 6 involved video visits [46,49,51,52,54,60], 5 focused on mobile health [45,48,53,59,63], 3 studied patient portals [58,61,62], and 1 on internet use for health purposes [55]. The category “Broad or mixed eHealth” was applied to studies that referred to eHealth in a broad way without specifying a single technology, or that combined multiple eHealth tools (eg, patient portals, telemonitoring, and mobile apps) without focusing on a single type.

#### Types of Interventions and Primary Outcome Measures (Intervention)

##### Overview

We categorize primary outcome measures of intervention success into 4 domains: adoption, use, skills, and attitudes. Adoption refers to initial uptake, such as first-time use of patient portals. Use captures actual engagement, including usage frequency. Skills encompass digital and eHealth literacy for navigation. Attitudes reflect user perceptions, such as confidence, comfort, and views on support. Table 4 shows the outcomes of the intervention studies across the domains. A “+” indicates a statistically significant positive effect, while “0” indicates that no statistically demonstrated effect was reported (including studies with nonsignificant results or studies that did not conduct statistical testing). The results of the strategy studies are also categorized in Multimedia Appendix 6 [26-44].

**Table 4.** Support actors involved in targeted eHealth interventions (n=21) included in this scoping review (2014‐2024) on support strategies and interventions for patients’ eHealth inclusion. The table categorizes proposed actors across micro- (interpersonal), meso- (technology supplier, health care organization, educational organization, and community organization), and macrolevel (policy) and links them to the primary outcome measures, categorized by adoption, use, skills, and attitudes.

Primary outcome measures and studies			Effect	Support actor							
				Interpersonal	Technology supplier		Health care organization	Educational organization		Community organization	Policy level
					Hardware	Software		Commercial	Public		
Adoption											
Portal log-in (yes or no)											
Lyles et al [58]			0^a^			✓^b^	✓				
Amount of portal enrollment activations											
Ramsey et al [61]			0				✓				
Rodriguez et al [62]			0				✓				
Use											
Frequency of technology use											
Drazich et al [50]			+^c^				✓		✓	✓	
Video visit completion											
Chu et al [49]			0				✓				
Gusdorf et al [51]			+				✓				
Mechanic et al [60]			+				✓				
Williams et al [42]			0				★^d^				
Worster et al [65]			+				✓				
Skills											
Digital literacy											
Alon et al [45]			+			✓	✓			✓	✓
Camacho and Torous [48]			+			✓	✓			✓	
Hernandez et al [53]			0	✓			✓				
eHealth literacy											
Bevilaqua et al [47]			+			✓			✓		
Lee and Kim [56]			+						✓		
Telehealth competence											
Taylor et al [64]			+			✓					
Attitudes											
Confidence in using technology											
Senteio et al [63]			+	✓						✓	
Cognitive load reduction											
Antonio et al [46]			0				✓			✓	
Comfort using technology											
Hawley et al [52]			0				✓		✓		
Primary qualitative findings											
Lessons from support interventions											
Hoffman et al [54]			Qual^e^		✓		✓				
Lim et al [57]			Qual		✓		✓	✓		✓	✓
Perceived value going online											
Jones et al [55]			Qual							✓	✓
Factors influencing the use of eHealth related to infomediaries											
Maliwichi and Chigoni [59]			Qual	✓	✓					✓	
Total				3	3	5	15	1	4	8	3

a 0: indicates outcomes for which no statistically demonstrated effect was reported (including nonsignificant findings and studies without inferential statistical testing).

b ✓: support actor involved.

c +: statistically significant improvement in the reported outcome.

d ★: Williams and colleagues’ study [42], which is primarily classified as a strategy study and therefore not included in the intervention count; it is nevertheless displayed here because, although its main focus was on implementation strategies, it also piloted support options that overlapped with intervention characteristics.

e Qual: qualitative study (no statistical test).

##### Outcomes Adoption

A total of 3 studies examined the effect of health care provider support on patient portal adoption [58,61,62]. Of this, 2 studies relied solely on health care actors: one assigned trained staff to educate older adults and assist with sign-ups during clinical visits [61], the other used a digital health navigator to help underserved patients enroll [62]. While both approaches increased sign-ups, neither of these 2 studies demonstrated statistically confirmed improvements in activation rates, and increases in portal engagement were reported descriptively without statistical testing.

In the third study, activation was tested but did not improve significantly [58]. This study compared in-person support with self-guided online tutorials by technology suppliers. It found no difference in activation rates, but did report significant improvements in self-reported eHealth literacy. This underlines the interventions’ value for skills, even though there was no significant effect on adoption [58]. All 3 studies identified sustainability challenges, such as resource limitations and the need for more intensive, long-term interventions for meaningful portal use.

##### Outcomes Use

The 6 studies on eHealth use all involve support solely from health care providers, without collaborations with other actors [49-51,60,65]. For use, 4 support interventions reported significant positive effects [50,51,60,65]. While adoption outcomes center on portal log-ins and activations, use-focused studies measure outcomes, including video visit completion. For instance, a study used a Telehealth Task Force, leading to significantly higher video visit completion rates [65]. The combined strategy and intervention study (included in the count of the strategy studies), which included in-person tech visits, user guides, and postvisit check-in texts, increased video visit completion by 15%, but lacked statistical tests [42].

##### Outcomes Skills

Interventions aimed at improving digital and eHealth literacy often involve multiple support actors, including collaborations between health care providers and community organizations [45,48], health care organizations and interpersonal contacts [53], and software suppliers and educational organizations [47]. A total of 2 studies examined the cross-domain collaborative Digital Outreach for Obtaining Resources and Skills (DOORs) program, an optional community service initiative in which partners from community organizations and health care providers collaborate to provide support to nonusers of eHealth [45,48]. These interventions, conducted at various locations including healthcare facilities and libraries, demonstrated significant improvements in digital skills. Additionally, studies pairing university students with older adults to enhance eHealth skills also reported significant positive effects on eHealth literacy [47,56]. In contrast, a study combining health care provider support with interpersonal contacts did not show significant improvements in digital literacy [54].

##### Outcomes Attitudes

Attitude-related findings were extracted from both quantitative and qualitative studies, with varying primary outcome measures. A total of 3 quantitative studies focused on varying primary outcome measures, including confidence using technology, cognitive load reduction, and comfort with technology [46,52,63]. Only the first study showed a significant positive effect, through an increase in confidence following intergenerational support within community organizations [63]. The other 2 studies, partly health care–based, did not report significant effects. The remaining 4 studies used qualitative methods exploring attitudes or factors and are indicated in white, as they did not assess significance [54,55,57,59]. One qualitative study launched a “Telehealth Team” in a hospital, supporting the transition to video visits with MyChart, offering help calls, technical troubleshooting, and patient preparation. Patients reported high satisfaction, though improvements were suggested, such as enabling proxy-user access and involving interpreters [61]. Another qualitative study highlighted the importance of approaching people in their native language, adjusting teaching pace, and focusing on font size and screen contrast for better support [57].

### Country-Level Analysis

We analyzed studies by country and health system (see Multimedia Appendix 3 [26-62] for the overview of included studies by country and health system characteristics). Most studies were conducted in the United States (21/40), followed by Asia (8/40; South Korea, China, Israel [Western Asia], and Singapore), Europe (5/40; Italy, Germany, the Netherlands, and the United Kingdom), Africa (1/40; Malawi), Canada (2/40), and Oceania (1/40; Australia). A total of 2 studies had a multicountry design: one in Canada and the United Kingdom, and another spanning India, Kenya, several West African countries, and Tanzania.

Across regions, some patterns emerged. US studies reported health care–provided support in 16/21 cases [33,38,42,45,48-54,58,60-62,65]. Among Asian studies, 5/8 emphasized interpersonal support [29,30,34,35,43], 2/8 emphasized community support [36,57], and 2/8 focused on policy-level support [44,57]. European studies showed a mix of support actors, including health care provider support as well as educational and community organization support [26,32,41,47,55]. The single Australian study highlighted family and friends as central intermediaries [31]. In Africa, the Malawi study described a community-driven intervention [59], while the multicountry African study examined public initiatives aimed at addressing community needs across India, Kenya, West Africa, and Tanzania [39].

### Relation Between Strategy Studies and Interventions: Actor Involvement

A total of 2 discrepancies were identified between strategy and intervention studies regarding actor involvement. Interpersonal support appeared in only 14.3% (3/21) of interventions, though it was proposed in 57.9% (11/19) of strategies. While interpersonal support can be beneficial, authors indicated it could lead to dependency rather than fostering autonomous eHealth use. A study incorporating health care support, followed by interpersonal support, if necessary, did not find significant improvements in digital literacy outcomes [53].

Health care providers engaged in 71.4% (15/21) of intervention studies but only 36.8% (7/19) of strategy studies. Interventions involving solely healthcare support showed no statistically demonstrated effects on adoption outcomes. Some of these interventions were prompted by the need to transition to eHealth during the COVID-19 pandemic [50]. Several studies also emphasized the need for sustainable funding beyond the project’s duration [60,62].

For other actors, such as community and policy-level participants, the differences between strategies and interventions were less pronounced.

## Discussion

### Principal Findings

This scoping review mapped evidence on support strategies and interventions to enhance eHealth inclusion. Across 19 strategy studies, 6 support actor types were identified: interpersonal contacts, technology suppliers, health care organizations, educational institutions, community organizations, and policymakers. Interpersonal contacts were the most frequently proposed actors, followed by community and health care organizations. The 21 intervention studies targeted 4 primary outcome measures: adoption, use, skills, and attitudes. Interventions solely involving health care organizations showed no statistically demonstrated effects on adoption, while use-focused interventions led by health care yielded mixed results, involving positive outcomes for 4 interventions. Attitude-focused interventions yielded mixed results. Multiactor collaborations showed statistically significant improvements in skills.

This review makes 5 research contributions. First, it identified a discrepancy: while interpersonal contacts were proposed in 58% (11/19) of strategy studies, only 14% (3/21) of intervention studies involved them. Strong ties with family and friends may appear to be a logical choice for support provision, given that they are often associated with high levels of trust [66]. However, research on self-management indicates that weak ties—such as those with organizations—tend to provide greater long-term durability, while focusing on strong ties could result in dependency [67]. Future research could explore the effectiveness of organizational support in providing durable, independence-oriented interventions.

Second, health care actors were advised to offer support in 37% (7/19) of strategy studies but engaged in 71% (15/21) of intervention studies. This might reflect the urgency that health care actors perceived in adopting eHealth and the immediate need for support. Most of these interventions were in pilot phases, with 14 out of 21 occurring in response to or during COVID-19 (see Multimedia Appendix 7 [45-65]) when physical consultations were limited and remote care became essential. Sustaining health care–led support beyond the pilot phase may be challenging, which might partly be rooted in health care providers’ high workloads. Addressing workforce shortages is important [1], although adding eHealth support tasks could further increase workloads. Therefore, multiactor interventions, such as the multiactor DOORs program between health care and community organizations—implemented in 2 included studies [45,48]—might offer more sustainable and transferable alternatives. However, while several multiactor interventions show positive effects, results are mixed, and further research is needed to explore factors influencing long-term effects.

Third, our review showed intervention heterogeneity in terms of the types of eHealth addressed, the support actors involved, and the outcome measures. This high variability presents challenges for comparing studies and for developing scalable and generalizable support solutions, as the inconsistency in outcome measures complicates comparison across studies. This aligns with research on eHealth, where heterogeneity across study approaches also makes comparisons difficult [68,69]. Even though researchers like Eysenbach have advocated for standardized ways to evaluate eHealth [70], actual evaluations are often not based on these guidelines. Future research is needed into guidelines allowing comparisons while considering the flexibility of the field of eHealth, experiencing fast developments.

Fourth, individuals may respond differently to distinct types of support or learning formats, resulting in varying outcomes depending on the intervention setting and the target group. As past research has emphasized, there is no one-size-fits-all approach to eHealth support, contributing to the wide range of strategies and outcomes observed in literature [71]. Future research should explore possibilities for the development of sustainable eHealth support interventions that incorporate personalization to accommodate diverse learning styles.

Fifth, our findings suggest regional differences in how eHealth support is organized, shaped by contextual factors. For example, US studies more often reflected health care–based interventions, whereas European studies reported a broader mix of actors. Asian studies highlighted interpersonal and community involvement, while the 2 studies conducted in African settings described grassroots or public initiatives designed to meet community needs. These contextual findings underline the importance of examining how health system design and cultural context influence effective eHealth support. At the same time, interpretation must remain cautious: the evidence base is dominated by US studies, and observed regional patterns may partly reflect study design rather than systemic differences. Future research could focus more specifically on linking health system characteristics, economic and cultural factors to the types of support that are most effective in different contexts, and on developing strategies to translate these insights into practice.

This review has several limitations. The heterogeneity in study aims, methods, and outcomes complicates comparisons. A scoping review was chosen to account for this variability, enabling a broad mapping of evidence. Consequently, no quality assessment was performed. We excluded gray literature to ensure a reproducible focus on peer-reviewed evidence, thereby providing a solid basis for future research, although this may have led to the omission of relevant practice-based initiatives. None of the reported intervention effects were negative. While this may suggest eHealth support is beneficial, it could also reflect publication bias, as studies with negative results are less often reported. The Hawthorne effect may also play a role, where participants alter behavior simply due to being observed [72]. Both factors could contribute to overestimating the appearance of positive results. Furthermore, many interventions, especially those involving health care providers, are resource-intensive or dependent on short-term funding, raising concerns about long-term sustainability. Also, studies included in this review often lack reporting information on the actual reasoning behind the design of the support and actors involved, stressing a need for future research.

While this review maps different strategies and interventions for support in eHealth use, the actual reported outcomes of interventions remain mixed, especially for adoption and attitudes. However, this review shows potential for health care organizations to establish support, as part of the interventions involving health care actors did show positive effects on eHealth inclusion. Health care organizations could benefit from external support networks or by integrating existing initiatives into healthcare practice. An example of this could be to explore opportunities for collaborations with community organizations or technology suppliers. However, sustaining positive effects requires both long-term funding and shared responsibility across actors. Further research is needed to determine which approaches yield sustainable, long-term improvements in eHealth inclusion.

### Conclusion

This scoping review aimed to identify strategies and interventions to enhance eHealth inclusion. Using a novel approach that combined the PIO and NASSS frameworks, we extracted and analyzed data across 40 studies. Strategy studies highlighted interpersonal contacts, technology suppliers, health care organizations, educational organizations, community organizations, and policymakers as key support actors. Although studies had a wide variety of methods used, primary outcomes targeted, and results found, some conclusions can be drawn to inform eHealth support. While many strategies focused on interpersonal contacts at the microlevel—such as those of family and friends—health care providers emerged as primary actors in targeted interventions. Their sole support was offered in interventions targeting adoption and use, yet research yielded mixed results for adoption. Support for improving eHealth skills often benefited from collaborative efforts involving educational organizations, community networks, and interpersonal support.

## Supplementary material

10.2196/79760 Multimedia Appendix 1 Search queries.

10.2196/79760 Multimedia Appendix 2 Overview data extraction items.

10.2196/79760 Multimedia Appendix 3 Overview of included studies by country and health system characteristics.

10.2196/79760 Multimedia Appendix 4 Description of strategy studies.

10.2196/79760 Multimedia Appendix 5 Description of intervention studies.

10.2196/79760 Multimedia Appendix 6 Results of strategy studies.

10.2196/79760 Multimedia Appendix 7 Results of intervention studies.

10.2196/79760 Checklist 1 PRISMA-ScR (Preferred Reporting Items for Systematic reviews and Meta-Analyses extension for Scoping Reviews) checklist.

## Abbreviations

CMB: Central Medical Library
DOORs: Digital Outreach for Obtaining Resources and Skills
NASSS: nonadoption, abandonment, scale-up, spread, and sustainability
PHR: personal health record
PIO: population, intervention, and outcome
PRISMA-ScR: Preferred Reporting Items for Systematic Reviews and Meta-Analyses extension for Scoping Reviews
RQ: research question
