# Supplementary material for: Support Strategies and Interventions for eHealth Inclusion: Scoping Review
Source: J Med Internet Res. 2025 Dec 12;27:e79760. doi: 10.2196/79760 (PMC12700317; doi:10.2196/79760)
Supplement: Multimedia Appendix 1 [file jmir-v27-e79760-s001.docx]

**Search queries: PubMed, Embase, Scopus, Web of Science**

- 1. **Search query PubMed**

For PubMed the following search query was used based on the PIO:

(“Patients”[Mesh] OR "Patient*"[tiab] OR "People"[tiab] OR "Client*"[tiab] OR “care consumer*”[tiab] OR “care receiver*”[tiab] OR “older adult*”[tiab])

**AND**

(((“Social Support”[Mesh] AND “Intervention*”[tiab]) OR (“Social support”[Mesh] AND “Structure*”[tiab]) OR (“Social support”[Mesh] AND “Network”[tiab]) OR (“Social support”[Mesh] AND “Relation*”[tiab]) OR (“Social support”[Mesh] AND “System”[tiab])) OR ((“Community Support”[Mesh] AND “Intervention*”[tiab]) OR (“Community Support”[Mesh] AND “Structure*”[tiab]) OR (“Community Support”[Mesh] AND “Network”[tiab]) OR (“Community Support”[Mesh] AND “Relation*”[tiab]) OR (“Community Support”[Mesh] AND “System”[tiab])) OR ((“Family Support”[Mesh] AND “Intervention*”[tiab]) OR (“Family Support”[Mesh] AND “Structure*”[tiab]) OR (“Family Support”[Mesh] AND “Network”[tiab]) OR (“Family Support”[Mesh] AND “Relation*”[tiab]) OR (“Family Support”[Mesh] AND “System”[tiab])) OR ((“Social Support”[Tiab] AND “Intervention*”[tiab]) OR (“Social support”[Tiab] AND “Structure*”[tiab]) OR (“Social support”[Tiab] AND “Network”[tiab]) OR (“Social support”[Tiab] AND “Relation*”[tiab]) OR (“Social support”[Tiab] AND “System*”[tiab])) OR ((“Community Support”[Tiab] AND “Intervention*”[tiab]) OR (“Community Support”[Tiab] AND “Structure*”[tiab]) OR (“Community Support”[Tiab] AND “Network”[tiab]) OR (“Community Support”[Tiab] AND “Relation*”[tiab]) OR (“Community Support”[Tiab] AND “System*”[tiab])) OR ((“support*”[tiab] AND “intervention*”[tiab]) OR (“support*”[tiab] AND “structure*”[tiab]) OR (“support*”[tiab] AND “network”[tiab]) OR (“support*”[tiab] AND “relation*”[tiab]) OR (“support*”[tiab] AND “system”[tiab])) OR (“training”[tiab] AND “intervention*”[tiab]) OR ((“help*”[tiab] AND “intervention*”[tiab]) OR (“help*”[tiab] AND “network”[tiab]) OR (“help*”[tiab] AND “relation*”[tiab]) OR (“help*”[tiab] AND “structure*”[tiab]) OR (“help*”[tiab] AND “system”[tiab])) OR ((“assist*”[tiab] AND “intervention*”[tiab]) OR (“assist*”[tiab] AND “network”[tiab]) OR (“assist*”[tiab] AND “relation*”[tiab]) OR (“assist*”[tiab] AND “structure*”[tiab]) OR (“assist*”[tiab] AND “system”[tiab])))

**AND**

("Telemedicine"[Mesh] OR “telemedicine”[tiab] OR “telehealth”[tiab] OR “telemonitoring”[tiab] OR “tele-monitoring”[tiab] OR “Digital health*”[tiab] OR “ehealth”[tiab] OR “e health”[tiab] OR “mhealth”[tiab] OR “mobile health”[tiab])

**AND**

("Health Literacy"[Mesh] OR “literac*”[tiab] OR “inclusion”[tiab] OR “divide”[tiab] OR “accessibility”[tiab])

**AND**

("2014/01/01"[Date - Publication] : "2024/12/31"[Date - Publication])

- 1. **Search query Embase**

For Embase the following search query was used based on the PIO:

('patient'/exp OR 'patient*':ab,ti,kw OR 'people':ab,ti,kw OR 'client*':ab,ti,kw OR 'care consumer*':ab,ti,kw OR 'care receiver*':ab,ti,kw OR 'older adult*':ab,ti,kw)

**AND**

('social support'/exp OR 'community support'/exp OR 'family support'/exp OR 'social support':ab,ti,kw OR 'community support':ab,ti,kw OR 'family support':ab,ti,kw OR 'support*':ab,ti,kw OR 'training*':ab,ti,kw OR 'help*':ab,ti,kw OR 'assist*':ab,ti,kw)

**AND**

('intervention*':ab,ti,kw OR 'structure*':ab,ti,kw OR 'network':ab,ti,kw OR 'relation*':ab,ti,kw OR 'system':ab,ti,kw)

**AND**

('telemedicine'/exp OR 'telemedicine':ab,ti,kw OR 'telehealth':ab,ti,kw OR 'telemonitoring':ab,ti,kw OR 'tele-monitoring':ab,ti,kw OR 'digital health*':ab,ti,kw OR 'ehealth':ab,ti,kw OR 'e health':ab,ti,kw OR 'mhealth':ab,ti,kw OR 'mobile health':ab,ti,kw) AND ('health literacy'/exp OR 'literac*':ab,ti,kw OR 'inclusion':ab,ti,kw OR 'divide':ab,ti,kw OR 'accessibility':ab,ti,kw)

- 1. **Search query Scopus (2014-2024 filter)**

For Scopus the following search query was used based on the PIO:

(TITLE-ABS-KEY (({Patients} OR {Patient*} OR {People} OR {Client*} OR {care consumer*} OR {care receiver*} OR {older adult*})

**AND**

(TITLE-ABS-KEY (({Social Support} AND {Intervention*}) OR ({Social support} AND {Structure*}) OR ({Social support} AND {Network}) OR ({Social support} AND {Relation*}) OR ({Social support} AND {System}) OR({Community Support} AND {Intervention*}) OR ({Community Support} AND {Structure*}) OR ({Community Support} AND {Network}) OR ({Community Support} AND {Relation*}) OR ({Community Support} AND {System}) OR({Family Support} AND {Intervention*}) OR ({Family Support} AND {Structure*}) OR ({Family Support} AND {Network}) OR ({Family Support} AND {Relation*}) OR ({Family Support} AND {System}) OR ({Social Support} AND {Intervention*}) OR ({Social support} AND {Structure*}) OR ({Social support} AND {Network}) OR ({Social support} AND {Relation*}) OR ({Social support} AND {System*}) OR ({Community Support} AND {Intervention*}) OR ({Community Support} AND {Structure*}) OR ({Community Support} AND {Network}) OR ({Community Support} AND {Relation*}) OR ({Community Support} AND {System*}) OR (support* AND {intervention*}) OR (support* AND {structure*}) OR (support* AND {network}) OR (support* AND {relation*}) OR (support* AND {system}) OR (training AND {intervention*}) OR (help* AND {intervention*}) OR (help* AND {network}) OR (help* AND {relation*}) OR (help* AND {structure*}) OR (help* AND {system}) OR (assist* AND {intervention*}) OR (assist* AND {network}) OR (assist* AND {relation*}) OR (assist* AND {structure*}) OR (assist* AND {system}))))

**AND**

(TITLE-ABS-KEY (({Telemedicine} OR {telemedicine} OR {telehealth} OR {telemonitoring} OR {tele-monitoring} OR {Digital health*} OR {ehealth} OR {e health} OR {mhealth} OR {mobile health})

**AND**

(TITLE-ABS-KEY({Health Literacy} OR {literac*} OR {inclusion} OR {divide} OR {accessibility}))

- 1. **Search query Web of Science**

For Web of Science the following search query was used based on the PIO:

(TS=(Patients OR Patient* OR People OR Client* OR care consumer* OR care receiver* OR older adult*))

**AND**

(TS=(Social Support AND Intervention*) OR TS=(Social support AND Structure*) OR TS=(Social support AND Network) OR TS=(Social support AND Relation*) OR TS=(Social support AND System) OR TS=(Community Support AND Intervention*) OR TS=(Community Support AND Structure*) OR TS=(Community Support AND Network) OR TS=(Community Support AND Relation*) OR TS=(Community Support AND System) OR TS=(Family Support AND Intervention*) OR TS=(Family Support AND Structure*) OR TS=(Family Support AND Network) OR TS=(Family Support AND Relation*) OR TS=(Family Support AND System) OR TS=(Social Support AND Intervention*) OR TS=(Social support AND Structure*) OR TS=(Social support AND Network) OR TS=(Social support AND Relation*) OR TS=(Social support AND System*) OR TS=(Community Support AND Intervention*) OR TS=(Community Support AND Structure*) OR TS=(Community Support AND Network) OR TS=(Community Support AND Relation*) OR TS=(Community Support AND System*) OR TS=(support* AND intervention*) OR TS=(support* AND structure*) OR TS=(support* AND network) OR TS=(support* AND relation*) OR TS=(support* AND system) OR TS=(training AND intervention*) OR TS=(help* AND intervention*) OR TS=(help* AND network) OR TS=(help* AND relation*) OR TS=(help* AND structure*) OR TS=(help* AND system) OR TS=(assist* AND intervention*) OR TS=(assist* AND network) OR TS=(assist* AND relation*) OR TS=(assist* AND structure*) OR TS=(assist* AND system))

**AND**

(TS=(Telemedicine OR telemedicine OR telehealth OR telemonitoring OR tele-monitoring OR Digital health* OR ehealth OR e health OR mhealth OR mobile health))

**AND**

(TS=(Health Literacy OR literac* OR inclusion OR divide OR accessibility))
