# Supplementary material for: Support Strategies and Interventions for eHealth Inclusion: Scoping Review
Source: J Med Internet Res. 2025 Dec 12;27:e79760. doi: 10.2196/79760 (PMC12700317; doi:10.2196/79760)
Supplement: Multimedia Appendix 2 [file jmir-v27-e79760-s002.docx]

|  | Strategy studies | | Intervention studies | |
| --- | --- | --- | --- | --- |
|  | Descriptives | Findings | Descriptives | Findings |
| Author(s), Year, Origin | X |  | X |  |
| Methods | X |  | X |  |
| Target group (P) | X |  | X |  |
| Sample size | X |  | X |  |
| Type of eHealth (P) | X |  | X |  |
| Purpose study | X |  |  |  |
| Adopted framework | X |  |  |  |
| Intended value (I) |  | X |  | X |
| Outcome measure |  |  | X |  |
| Project (I) |  |  | X |  |
| Project content (I) |  |  | X |  |
| (Co)users (I) |  | X |  | X |
| Support actors (I) |  |  |  | X |
| Initiating organization (I) |  |  |  | X |
| External stakeholders (I) |  | X |  | X |
| Legislative context (I) |  | X |  | X |
| Temporal dynamics (I) |  | X |  | X |
| Realized value (I/O)* |  | X |  | X |
| Future intentions (I/O)* |  | X |  | X |
| **In the strategy studies, realized value and future intentions were specified, but these did not result from a support intervention as defined in our PIO. Instead, these items focus on the provision of support itself, where the outcome is the support (I). In contrast, for intervention studies, the outcome is the effect of the support (O).* | | | | |
