# Supplementary material for: Support Strategies and Interventions for eHealth Inclusion: Scoping Review
Source: J Med Internet Res. 2025 Dec 12;27:e79760. doi: 10.2196/79760 (PMC12700317; doi:10.2196/79760)
Supplement: Multimedia Appendix 4 [file jmir-v27-e79760-s004.docx]

| **Table S1.** Descriptions of strategy studies. | | |  |  |  |  |
| --- | --- | --- | --- | --- | --- | --- |
| **1^st^ Author, Year, Origin** | **Type of eHealth** | **Purpose/Intended value** | **Target group** | **Methods** | **Sample size** | **Framework** |
| Arighi [26], 2021, Italy | Video visits | Understand which factors can influence the success of video visits. | Patients, dementia | Quantitative | n = 108 patients participated with cognitive impairment. | NA: data successfulness video visit, sociodemographic data on age, and data on the presence of a caregiver |
| Chen [27], 2022, USA | eHealth, in general | Explore experiences of physicians in eHealth with older adults during the COVID-19 pandemic and identify strategies to overcome barriers to use eHealth for elderly. | Primary Care Physicians and geriatricians on older adults >65 | Qualitative | n = 15 PCPs and n = 33 geriatricians participated. | NS: “a survey based on ‘recent literature’” |
| Curran [28], 2023, Canada | eHealth, in general | Exploring patients’ experiences and perspectives with adoption and use of eHealth during COVID-19.  Identify educational and informational needs of patients to inform future strategies for supporting patients. | Patient representatives representing 3-4 care regions | Qualitative | n = 5 patient representatives represented 3/4 care regions in the province. | Roger’s Innovation Diffusion Theory62 |
| Han [29], 2021, South Korea | eHealth, in general | Verify what personal and social factors influence older adults in using eHealth and suggest digital training and supportive environment plans to bridge the digital divide among older adults. | Older adults | Quantitative | n = 1,662 participants in the survey. | TAM-3 theoretical framework |
| Hayat [30], 2017, Israel | eHealth, in general | Examine how the social ties interplay with respondents’ eHealth literacy and perceived health outcomes. | Adults >21 | Quantitative | n = 819 participants. | Social capital and eHealth literacy framework |
| Hodge, [31] 2017, Australia | Internet, including for health | Examine how user patterns by consumers and service providers address or widen the digital divide for older people. | Older adults >60 and service providers | Qualitative | n = 6 older adult internet users and n = 10 service providers | Social network mapping |
| Jokisch [32], 2022, Germany | eHealth, in general | Examining relations of technology acceptance, including support seeking (family, informal, formal/institutional) to older adults’ intention to adopt eHealth services. | Older adults >50 | Quantitative | n = 478 older adults included in the survey. | Extended TAM: support (family, informal, formal), self-efficacy, perceived usefulness, privacy concerns, ICT knowledge |
| Khairat [33], 2023, USA | eHealth, in general | Explore eHealth use and obtain actionable recommendations to improve user experience from patients and providers. | Patients aged >18 with ≥1 chronic disease and primary care providers | Qualitative | n = 65 patients and n = 21 primary care providers were included. | Benson Framework |
| Kim [34], 2024, South Korea | Mobile health applications | Investigate whether social support affects mHealth use through eHealth literacy and whether/how the strength of the mediated effect through eHealth literacy varies with differing levels of app design importance. | Older adults >65 having diabetes | Quantitative | n = 180 participants responded to the survey, of which n = 133 responses were valid. | Multidimensional Scale of Perceived Social Support, Korean version eHealth Literacy Scale, Dimensions of aesthetics and functionality |
| Lee [35], 2024, South Korea | eHealth, in general | Examine the relationships among eHealth literacy, self-efficacy, social support and self-management in people with diabetes type 2· | Patients >19 having diabetes | Quantitative | n = 453 participants. | Condition-specific eHealth literacy scale, Diabetes management self-efficacy scale, Medical Outcomes Study Social Support Survey, Diabetes Self-management Scale |
| Lin [36], 2021, China | eHealth, in general | Explore the eHealth literacy, health knowledge and health behavior of older adults and investigate relationships with use of library or community activities and eHealth literacy. | Older adults >45 | Quantitative | n = 215 completed survey responses. | Health conditions, library utilization, eHealth literacy scale, health knowledge test, daily health-related activities |
| Marston [37], 2019, UK and Canada | eHealth, in general | Examine the experiences of older adults (65+) with technology, exploring how they embraced various types of ICT, and what challenges were faced in the take-up. | Older adults >65 | Qualitative | n = 37 participants were included, of which n = 16 are Canadian and n = 21 are from the UK. n = 20 of the 37 are from rural settings and n = 17 from urban ones. | Previously used survey covering: Technology use; (2) Internet ownership and use; (3) Social networking; (4) Digital device ownership; (5) Purchasing patterns; (6) Quantified Self and lifelogging; (7) Information sharing and privacy issues; and (8) Demographics. Focus groups: ICT use/ownership; rationale for using ICTs; social media habits and perceptions; privacy issues and concerns; sharing of information (e.g., why, how, type of content shared), from a traditional (pen/paper) and digital (mobile app/Facebook) standpoint; and what the future holds for ICT in society, health, and ageing populations |
| Pack [38], 2024, USA | Patient portals and video visits | Gain insights into telehealth use and needs among CHC patient participants. | Patients >18 (in surveys and interviews) | Mixed methods | n = 500 completed surveys and n = 24 interview respondent, including 12 users and 12 non-users. | Socio-demographic characteristics, Health Literacy 6 |
| Radovanovic, 2020 [39], India, Kenya, West Africa, Tanzania | eHealth, in general | Identify KPIs for sustainable development related to digital literacy | Adults, Africa and India in variety of case studies | Mixed methods | Various case studies and varying sample sizes were discussed. | NS |
| Shahid [40], 2023, Canada | eHealth, in general | Outline experiences of health care organizations rapidly implementing eHealth during the first wave of COVID-19 and examine the consideration of health equity. | Patients over and under the age of 65 (interviews at multiple sites) | Qualitative | n = 39 interviews were conducted at n = 4 different sites | Conceptual framework of access to health care |
| Van Middelaar [41], 2018, the Netherlands | Internet platform cardiovascular self-management | Assess which factors influence initial and sustained platform engagement. Assess older people’s views on implementation of the platform in the primary care setting. | People >65 years old, with an increased risk of cardiovascular diseases | Qualitative | 17 interviews with n = 20 participants | NS: questions on experiences platform |
| Williams [42], 2021, USA | eHealth for chronic disease management | Describe early implementation challenges and stakeholder driven process adaptations specific to the digitally delivered chronic disease care management strategy | People >21 years old with >2 chronic conditions, patient partners, and care managers | Quantitative | n = 9 patient partners, n = 129 participants and n = 32 care managers | NS: mechanisms to capture feedback: study support log, implementation meeting minutes, case interview log, patient partners work group log |
| Wu [43], 2023, China | eHealth, in general | Explore potential mechanisms between self-management, social support, eHealth literacy, and self-efficacy. | Older people >60 with >1 chronic disease | Quantitative | n = 289 survey respondents of community home-based elderly | Chronic disease self-management study measures, Social support rate scale, eHealth literacy scale, general self-efficacy scale |
| Zhao [44], 2023, China | eHealth, in general | Explore how interactions between older adults and the outside world shape eHealth use and might narrow the age-based digital divide. | Older hospital patients >60 and their family members | Qualitative | n = 12 patients and n = 21 family members | Narratives of key events marking development of digital capabilities, allowing to compare digital capability levels and generate a process model for digital capability development |

**References**

26. Arighi A, Fumagalli GG, Carandini T, et al. Facing the digital divide into a dementia clinic during COVID-19 pandemic: caregiver age matters. Neurol Sci. Apr 2021;42(4):1247-1251. [doi: 10.1007/s10072-020-05009-w] [Medline: 33459891]

27. Chen K, Davoodi NM, Strauss DH, et al. Strategies to ensure continuity of care using telemedicine with older adults during COVID-19: a qualitative study of physicians in primary care and geriatrics. J Appl Gerontol. Nov 2022;41(11):2282-2295. [doi: 10.1177/07334648221109728] [Medline: 35711106]

28. Curran VR, Hollett A, Peddle E. Patient experiences with virtual care during the COVID-19 pandemic: phenomenological focus group study. JMIR Form Res. May 1, 2023;7:e42966. [doi: 10.2196/42966] [Medline: 37036827]

29. Han S, Nam SI. Creating supportive environments and enhancing personal perception to bridge the digital divide among older adults. Educ Gerontol. Aug 3, 2021;47(8):339-352. [doi: 10.1080/03601277.2021.1988448]

30. Hayat TZ, Brainin E, Neter E. With some help from my network: supplementing eHealth literacy with social ties. J Med Internet Res. Mar 30, 2017;19(3):e98. [doi: 10.2196/jmir.6472] [Medline: 28360024]

31. Hodge H, Carson D, Carson D, Newman L, Garrett J. Using internet technologies in rural communities to access services: the views of older people and service providers. J Rural Stud. Aug 2017;54:469-478. [doi: 10.1016/j.jrurstud.2016.06.016]

32. Jokisch MR, Schmidt LI, Doh M. Acceptance of digital health services among older adults: findings on perceived usefulness, self-efficacy, privacy concerns, ICT knowledge, and support seeking. Front Public Health. 2022;10:1073756. [doi: 10.3389/fpubh.2022.1073756] [Medline: 36582385]

33. Khairat S, Chourasia P, Muellers KA, Andreadis K, Lin JJ, Ancker JS. Patient and provider recommendations for improved telemedicine user experience in primary care: a multi-center qualitative study. Telemed Rep. 2023;4(1):21-29. [doi: 10.1089/tmr.2023.0002] [Medline: 36950478]

34. Kim M, Kim B, Park S. Social support, eHealth literacy, and mHealth use in older adults with diabetes: moderated mediating effect of the perceived importance of app design. Comput Inform Nurs. Feb 1, 2024;42(2):136-143. [doi: 10.1097/CIN.0000000000001081] [Medline: 38129323]

35. Lee EH, Lee YW, Kang EH, Kang HJ. Relationship between electronic health literacy and self-management in people with type 2 diabetes using a structural equation modeling approach. J Nurs Res. Jan 1, 2024;32(1):e315. [doi: 10.1097/jnr.0000000000000588] [Medline: 38128065]

36. Lin Z, Zhang Y, Matteson M, et al. Older adults’ eHealth literacy and the role libraries can play. J Lib Inf Sci. Sep 2021;53(3):488-498. [doi: 10.1177/0961000620962847]

37. Marston HR, Genoe R, Freeman S, Kulczycki C, Musselwhite C. Older adults’ perceptions of ICT: main findings from the Technology In Later Life (TILL) study. Healthcare (Basel). Jul 4, 2019;7(3):86. [doi: 10.3390/healthcare7030086] [Medline: 31277387]

38. Pack AP, Rusca P, Llaneza J, et al. Optimizing telehealth services: a mixed-methods needs assessment conducted among community health center patients. Med Care. Jan 1, 2024;62(1):30-36. [doi: 10.1097/MLR.0000000000001932] [Medline: 37796220]

39. Radovanović D, Holst C, Belur SB, et al. Digital literacy key performance indicators for sustainable development. SI. 2020;8(2):151-167. [doi: 10.17645/si.v8i2.2587]

40. Shahid S, Hogeveen S, Sky P, et al. Health equity related challenges and experiences during the rapid implementation of virtual care during COVID-19: a multiple case study. Int J Equity Health. Mar 11, 2023;22(1):44. [doi: 10.1186/s12939-023-01849-y] [Medline: 36906566]

41. van Middelaar T, Beishuizen CRL, Guillemont J, et al. Engaging older people in an internet platform for cardiovascular risk self-management: a qualitative study among Dutch HATICE participants. BMJ Open. Jan 21, 2018;8(1):e019683. [doi: 10.1136/bmjopen-2017-019683] [Medline: 29358447]

42. Williams K, Markwardt S, Kearney SM, et al. Addressing implementation challenges to digital care delivery for adults with multiple chronic conditions: stakeholder feedback in a randomized controlled trial. JMIR Mhealth Uhealth. Feb 1, 2021;9(2):e23498. [doi: 10.2196/23498] [Medline: 33522981]

43. Wu Y, Wen J, Wang X, et al. Chinese community home-based aging institution elders’ self-management of chronic non-communicable diseases and its interrelationships with social support, e-health literacy, and self efficacy: a serial multiple mediation model. Patient Prefer Adherence. 2023;17:1311-1321. [doi: 10.2147/PPA.S412125] [Medline: 37255948]

44. Zhao Y, Zhang T, Dasgupta RK, Xia R. Narrowing the age‐based digital divide: Developing digital capability through social activities. Information Systems Journal. Mar 2023;33(2):268-298. [doi: 10.1111/isj.12400]
