# Supplementary material for: Support Strategies and Interventions for eHealth Inclusion: Scoping Review
Source: J Med Internet Res. 2025 Dec 12;27:e79760. doi: 10.2196/79760 (PMC12700317; doi:10.2196/79760)
Supplement: Multimedia Appendix 5 [file jmir-v27-e79760-s005.docx]

| **Table S2. Descriptions of intervention studies.** | | | | | | | |
| --- | --- | --- | --- | --- | --- | --- | --- |
| **1^st^ Author, Year, Origin** | **Type of eHealth** | **Project** | **Project content** | **Target group** | **Methods** | **Sample size** | **Outcome Measurement** |
| Alon [45], 2023, USA | Mobile health: smartphones | Digital Outreach for Obtaining Resources and Skills (DOORs) | 8-week program covering 29 tasks during 90-minute helping sessions. Possibility of smartphone provision. | Adults: serious mental illnesses | Mixed methods | Session attendance fluctuations of n = 10 to n = 19 participants. Survey completion for n = 92 respondents. | Functional digital literacy questions and confidence-based, semi-structured interviews on experiences |
| Antonio [46], 2023, USA | Video visits | FQHC volunteers | Separate 10-30 minute helping sessions 3 eHealth tasks. | Patients: Vulnerable | Quantitative | Intervention completion for n = 34 participants. Survey completion for n = 128 respondents, of which n = 15 participated in the intervention. | Cognitive load measurement of use, Technology experience, Technology use frequency |
| Bevilacqua [47], 2021, Italy | eHealth in general | ACCESS | Online platform: 4-week program of 5 90-minute eHealth modules. | Older adults (> 50) | Quantitative | Program attendance of n = 58 participants. | Functional eHealth literacy scale score |
| Camacho [48], 2023, USA | Smartphones | Digital Outreach for Obtaining Resources and Skills (DOORs) | 8-week program covering 29 tasks during 90-minute helping sessions. One 45-minute lesson on app evaluation for participants in an IPU. | Clubhouse participants and IPU patients | Quantitative | Session attendance of n = 113 participants pre-intervention and n = 87 post-intervention. For the IPU, participant attendance was n = 74 pre-intervention and n = 52 post-intervention. | Self-reported digital literacy, Changes in functional skills |
| Chu [49], 2022, USA | Video visits | Video Visits for Elderly Patients (VVEP) | One time phone calls to walk people through a video visit. | Older adults (> 65) | Quantitative | Reach of n = 1,025 patients, of which n = 192 received technical assistance. | Amount of completed visits and type of visits |
| Drazich [50], 2021, USA | eHealth in general | Addressing the Digital Divide to  Improve Patient-Centered Outcomes Research (ADD2PCOR) | 2 sessions of group training and a 1-hour individual training session. Tablet provision. | Community stakeholders | Quantitative | Inclusion of n = 20 community stakeholders of which n = 16 completed the survey | Frequency of technology use, Functional Assessment of Comfort Employing Technology Scale |
| Gusdorf [51], 2023, USA | Video visits | Vanderbilt Telehealth Volunteer Program (VTVP) | Phone call assistance on five different eHealth topics. | Patients in general | Quantitative | Participation of n = 5182 patients, assisted by n = 135 medical students. | Likelihood of successful video visits via the amount of successful vs. failed video visits |
| Hawley [52], 2023, USA | Video visits | In-home team member support Veterans Affairs (VA) | Study team member observation pre-visit. In-home team member resolving challenges pre- and during visit. Possibility of device loan followed by a test visit. | Older adults (> 65), >2 chronic medical conditions, no dementia | Qualitative | Video visit completion by n = 20 participants of which n = 10 needed assistance before or during the visit. | Comfort using technology, Video visit completion, The Consolidated Framework for Implementation Research (CFIR), matching tool Expert Recommendations for Implementing Change (ERIC) |
| Hernandez-Ramos [53], 2021, USA | Mobile health: specific application | One-on-one staff-patient partnerships DIAMANTE diabetes app (DIAMANTE) | Individualized assistance based on a questionnaire. | Patients: limited digital literacy, English or Spanish speaking | Quantitative | Participation in intervention by n = 43 patients participated in the intervention. | Self-reported digital literacy, Rate of smartphone ownership, Proportion of patients digital literacy barriers |
| Hoffman et al [54], 2020, USA | Video visits | MyChart Genius Support Team and IT-team (MyChart Genius) | Phone call assistance on video visit usage. Technical troubleshooting for issues. | Patients in general | Quantitative | Responses of n = 10,422 patients to the survey. | % of conversion to video visits |
| Jones et al [55], 2015, UK | The Internet: including for health purposes | Plymouth Senior Net Volunteer support (PSN Volunteers) | Six small-group sessions of help over 12 hours or 8 sessions of one-on-one at-home support. | Older adults (> 65) | Quantitative | Recruitment of n = 144 people, of which n = 58 were supported at home and n = 86 in small group sessions | Number of contacts with others after at-home support in eHealth, perceived value of going online |
| Lee and Kim [56], 2019, USA | eHealth in general | Intergenerational Mentor-Up (IMU) | 276 mentoring support hours provided by seventy-eight undergraduate students. | Older adults (> 65) and undergraduate students | Mixed methods | In total n = 78 undergraduate students were paired with n = 55 seniors completing one-on-one technology tutorials. | eHealth literacy, Attitudes towards the Internet, Willingness to use online health information, Technophobia about computer use, social isolation |
| Lim et al [57], 2022, Singapore | eHealth in general | Project Wire Up | 3-month training program in six sessions. Smartphone provision. | Older adults (> 60): vulnerable and low SES | Qualitative | In total n > 300 adults have benefited from the program. | Perceived barriers to eHealth use and reduction in this, factors influencing use |
| Lyles et al [58], 2019, USA | Patient portals | MYSFHEALTH platform training (MYSFHEALTH) | Online platform: eleven how-to videos on learning platform. | Patients in general | Quantitative | Total of n = 93 participants enrolled in the trial. Portal use outcomes available for n = 88 individuals. Follow-up surveys completed by n = 75 individuals. | Portal sign-up rates, Amount of people newly web-enabled, eHealth literacy scale score, Self-reported interest in using the portal, Reported reasons for use |
| Maliwichi et al [59], 2022, Malawi | Mobile health: smartphones | Chipatala Cha Pa Foni (CCPF) | Intermediaries provide phone access by having custody of mobile phones that others may use. | Citizens: maternal healthcare clients | Qualitative | Inclusion of n = 20 maternal healthcare clients and n = 7 infomediaries | Reduction in challenges mobile phone use, factors influencing ehealth use |
| Mechanic et al [60], 2022, USA | Video visits | Patient Navigator Program (PNP) | One-time phone-based outreach by a navigator using a script covering the steps required for the patient to connect to their visit. | Adults: scheduled video visit, primary care and gerontology clinic | Quantitative | N = 4066 eligible scheduled video visits of which n = 3031 participated in the control group and n = 1035 in the intervention. | Differences in video visit success rate and canceled or no-show rate between intervention and comparator group. |
| Ramsey et al [61], 2018, USA | Patient portals | MyChart Genius | Support in examination rooms on portal education, privacy and functionality. | Patients (> 13) | Quantitative | N = 96 patients were approached. N = 84 agreed to sign up. N = 63 completed surveys. | Portal enrollment and activation, Satisfaction with project |
| Rodriguez et al [62], 2023, USA | Patient portals | Digital Health Navigators (DHN) | Trained staff member training on log-on to devices, portal functionalities. | Patients: Diabetes type 2 | Quantitative | N = 121 patients were enrolled in the program. | Outreach successes, Completing diabetes-related tasks on the portal, CFIR evaluation |
| Senteio et al [63], 2021, USA | Mobile health: smartphones | Intergenerational Technology Transfer (TTT) | An health education session to promote self-efficacy of technology. | Older adults (> 55) and younger peers in their network (18-54): African American | Qualitative | N = 39 older adults were included and n = 26 younger peers. | Self-management skills, Use of technology |
| Taylor et al [64], 2023, USA | eHealth, in general | Initial telehealth education modules (i-TEMs) | Online provision of five eHealth modules. | Older adults (> 60) | Mixed methods | Participation of n = 5 people in interviews, and of n = 94 prequestionnaire respondents, n = 53 respondents to post-questionnaires (56·4%). | Telehealth Competency Questionnaire-Consumer: thirteen item self-assessment |
| Worster et al [65], 2023, USA | eHealth, in general | Telehealth Task Force (TTF) | One-on-one education of various tasks. Possibility of smartphone provision. | Patients: Cancer | Quantitative | Pre-intervention n = 90 people included, during intervention n = 194, and post-intervention n = 271· | Successful Telehealth Access, Health Literacy |

**References**

45. Alon N, Perret S, Torous J. Working towards a ready to implement digital literacy program. Mhealth. 2023;9:32. [doi: 10.21037/mhealth-23-13] [Medline: 38023777]

46. Antonio MG, Williamson A, Kameswaran V, et al. Targeting patients’ cognitive load for telehealth video visits through student-delivered helping sessions at a United States federally qualified health center: equity-focused, mixed methods pilot intervention study. J Med Internet Res. Feb 1, 2023;25:e42586. [doi: 10.2196/42586] [Medline: 36525332]

47. Bevilacqua R, Strano S, Di Rosa M, et al. eHealth literacy: from theory to clinical application for digital health improvement. Results from the ACCESS training experience. Int J Environ Res Public Health. Nov 10, 2021;18(22):11800. [doi: 10.3390/ijerph182211800] [Medline: 34831555]

48. Camacho E, Torous J. Impact of digital literacy training on outcomes for people with serious mental illness in community and inpatient settings. Psychiatr Serv. May 1, 2023;74(5):534-538. [doi: 10.1176/appi.ps.20220205] [Medline: 36164771]

49. Chu JN, Kaplan C, Lee JS, Livaudais-Toman J, Karliner L. Increasing telehealth access to care for older adults during the COVID-19 pandemic at an academic medical center: Video Visits for Elders Project (VVEP). Jt Comm J Qual Patient Saf. Mar 2022;48(3):173-179. [doi: 10.1016/j.jcjq.2021.11.006] [Medline: 35027304]

50. Drazich BF, Nyikadzino Y, Gleason KT. A program to improve digital access and literacy among community stakeholders: cohort study. JMIR Form Res. Nov 10, 2021;5(11):e30605. [doi: 10.2196/30605] [Medline: 34757316]

51. Gusdorf RE, Shah KP, Triana AJ, et al. A patient education intervention improved rates of successful video visits during rapid implementation of telehealth. J Telemed Telecare. Sep 2023;29(8):607-612. [doi: 10.1177/1357633X211008786] [Medline: 33975506]

52. Hawley CE, Wagner C, Venegas MD, et al. Connecting the disconnected: leveraging an in‐home team member for video visits for older adults. J Am Geriatr Soc. May 2024;72(5):1408-1419. URL: <https://agsjournals.onlinelibrary.wiley.com/toc/15325415/72/5> [doi: 10.1111/jgs.18663] [Medline: 37960887]

53. Hernandez-Ramos R, Aguilera A, Garcia F, et al. Conducting internet-based visits for onboarding populations with limited digital literacy to an mHealth intervention: development of a patient-centered approach. JMIR Form Res. Apr 29, 2021;5(4):e25299. [doi: 10.2196/25299] [Medline: 33872184]

54. Hoffman PE, London YR, Weerakoon TS, DeLucia NL. Rapidly scaling video visits during COVID-19: The ethos of virtual care at Yale Medicine. Healthcare (Basel). Dec 2020;8(4):100482. [doi: 10.1016/j.hjdsi.2020.100482]

55. Jones RB, Ashurst EJ, Atkey J, Duffy B. Older people going online: its value and before-after evaluation of volunteer support. J Med Internet Res. May 18, 2015;17(5):e122. [doi: 10.2196/jmir.3943] [Medline: 25986724]

56. Lee OEK, Kim DH. Bridging the digital divide for older adults via intergenerational mentor-up. Res Soc Work Pract. Oct 2019;29(7):786-795. [doi: 10.1177/1049731518810798]

57. Lim HA, Lee JSW, Lim MH, et al. Bridging connectivity issues in digital access and literacy: reflections on empowering vulnerable older adults in Singapore. JMIR Aging. May 3, 2022;5(2):e34764. [doi: 10.2196/34764] [Medline: 35503520]

58. Lyles CR, Tieu L, Sarkar U, et al. A randomized trial to train vulnerable primary care patients to use a patient portal. J Am Board Fam Med. 2019;32(2):248-258. [doi: 10.3122/jabfm.2019.02.180263] [Medline: 30850461]

59. I Maliwichi P, Chigona W. Towards a framework on the use of infomediaries in maternal mHealth in rural Malawi. IJIKM. 2022;17:387-411. [doi: 10.28945/5015]

60. Mechanic OJ, Lee EM, Sheehan HM, et al. Evaluation of telehealth visit attendance after implementation of a patient navigator program. JAMA Netw Open. Dec 1, 2022;5(12):e2245615. [doi: 10.1001/jamanetworkopen.2022.45615] [Medline: 36480202]

61. Ramsey A, Lanzo E, Huston-Paterson H, Tomaszewski K, Trent M. Increasing patient portal usage: preliminary outcomes from the MyChart Genius Project. J Adolesc Health. Jan 2018;62(1):29-35. [doi: 10.1016/j.jadohealth.2017.08.029] [Medline: 29169768]

62. Rodriguez JA, Charles JP, Bates DW, Lyles C, Southworth B, Samal L. Digital healthcare equity in primary care: implementing an integrated digital health navigator. J Am Med Inform Assoc. Apr 19, 2023;30(5):965-970. [doi: 10.1093/jamia/ocad015] [Medline: 36795062]

63. Senteio CR, Hershey DS, Campbell T, Mandal S. Intergenerational technology transfer: enhancing African American older adults’ self-efficacy for diabetes self-management. Prog Community Health Partnersh. 2021;15(4):e5. [doi: 10.1353/cpr.2021.0048] [Medline: 34975018]

64. Taylor S, Souza S, Little L, Odiaga J. Enhancing telehealth competency: development and evaluation of education modules for older adults. OTJR (Thorofare N J). Jul 2023;43(3):478-486. [doi: 10.1177/15394492231153115] [Medline: 36757088]

65. Worster B, Waldman L, Garber G, et al. Increasing equitable access to telehealth oncology care in the COVID-19 national emergency: creation of a telehealth task force. Cancer Med. Feb 2023;12(3):2842-2849. [doi: 10.1002/cam4.5176] [Medline: 36210751]
