# Supplementary material for: Support Strategies and Interventions for eHealth Inclusion: Scoping Review
Source: J Med Internet Res. 2025 Dec 12;27:e79760. doi: 10.2196/79760 (PMC12700317; doi:10.2196/79760)
Supplement: Multimedia Appendix 6 [file jmir-v27-e79760-s006.docx]

| **Table S3. Results of strategy studies.** | | | | |  |  |  |  |  |
| --- | --- | --- | --- | --- | --- | --- | --- | --- | --- |
|  | **Population** | **Intervention** | | | | | | | |
| **Authors** | **Users** | **Organization: Support Site** | **Intended value** | **Realized outcomes** | | **External Stakeholders** | **Legislative Context** | **Temporal Dynamics** | **Future intentions/Proposed Strategy** |
| Arighi et al [26] | Patients, dementia | Hospital: Alzheimer Center | Understand which factors influence eHealth successfulness. | N = 74 patients successfully completed video visits while n = 34 failed. Age differences were not significant between groups. Patients were stratified by presence of a caregiver, and success rates were higher when a younger caregiver assisted: 80% success with a younger caregiver and 95% in case of both a same and younger generation caregiver present. | | NS | Italian parliament views digital skills as key competence. | COVID-19 caused the transformation to video visits | Need for **political and community interventions** aiming to solidify the **social safety net** to support socially vulnerable populations. |
| Chen et al [27] | PCPs and geriatricians caring for older adults (65+) | NA: recruitment surveys via social media | Identify concrete strategies PCPs and geriatricians employes to overcome barriers to using telehealth with older adults | Six major interview themes were identified. Important for support is advanced preparation of video visits by speaking to patients in advance, meeting patient needs through using multiple eHealth modalities, home-based support, reassurance opportunities, adapt to disabilities such as hearing issues by speaking more clearly, and involve family members and other caregivers. | | NS | NS | NS | Adapt to sensory needs. Prepare adults for visits in advance. **Include caregivers** and provide additional education and supportive resources. |
| Curran et al [28] | Patients, in general | Patient Advisory Council for Recruitment | Identify patients’ educational and informational needs of to inform future strategies for supporting patients' use of eHealth. | For patient education it is beneficial to have: ongoing education and support, knowledge on how information is protected, knowledge on how to prepare for eHealth use, be aware of multiple information outlets to address different people and their varying needs (via libraries, collaborations with communities etc.), focus on peer support. | | NS | Viewpoint: patients have a fundamental right to be engaged in research. | Patients had to have experienced eHealth during COVID-19 | Account for patients’ needs in providing support and focus on peer support. |
| Han and Nam [29] | Older adults (55-85 years old) | NA: Population-broad | Verify what factors influence older adults using technology and suggest digital training to bridge the digital divide among older adults. | Social support has a significant positive relation with the perceived usefulness of technology (b=0·064, p=0·022) and perceived ease of use (b=0·201, p=0·021). Social norm had no significant effect on perceived usefulness but had a positive significant effect of perceived ease of use (b=0·169, p=0·024). | | Ministry of Science and ICT South Korea | NS | Pre-COVID 19 | A variety of **formal support systems** should be established to help older adults encountering difficulties, as **social support** positively affects perceived ease of use and usefulness. Yet, informal support from family and friends is not always available. |
| Hayat et al [30] | Adults (21+) | NA: Population-broad | Examine how interpersonal resources such as social ties interplay with respondents’ eHealth literacy and perceived health outcomes | Interaction plot showed finding others with similar health concerns yields higher perceived health outcomes when eHealth literacy is low. There is a potential benefit of social ties in compensating for low eHealth literacy. | | Israeli Central Bureau of Statistics for stratifying the data. | NS | NS | Study shows the importance of considering **social ties and social capital** to enhance eHealth literacy. |
| Hodge et al [31] | Older Internet users (60+) and service providers | NA: Population-broad | Examine how patterns of use by consumers and service providers address or exacerbate the digital divide affecting older people. | Most connections in this case study were intermediated by family or friends, stressing the importance of social contacts in facilitating digital participation for elderly. Also, third party connections were seen as an important faciltator for use. | | NS | Government established ‘Digital Transfromation Office’ aiming to deliver all government services digitally | NS | Strategies to enhance **social inclusion** and access to stronger **social networks** are at least as important to address the digital divide as strategies to improve technological capabilities. |
| Jokisch et al [32] | Older adults (50+) | NA: Population-broad | Examining relations of technology acceptance, including support seeking (family, informal, formal/institutional) to older adults’ intention to adopt eHealth services. | Significant associations between: family support and intention to use eHealth services (b=0·16, p<0·001), formal support and intention to use eHealth (b=0·082, p<0·05), formal support and perceived usefulness (b=0·247, p<0·001). Informal support seeking and perceived usefulness had a marginal significant association (b=0·11, p=0·050). No significant relations between informal support and intention to use eHealth or perceived usefulness, and family support and perceived usefulness. | | Ministry of Socia Affairs and Integration: part of their larger study project | NS | NS | **Formal education** was rated as important and was associated with more favorable scores, indicating need for suitable education. **However,** people in the sample were high educated, and others might have trouble accessing these services. **Voluntary program**s can be needed to closely tailor to the needs of older people not feeling addressed in traditional courses. |
| Khairat et al [33] | Primary care patients (18+) | Three patient centered-networks in the region | Explore eHealth use and obtain actionable recommendations to improve user experience from patients and providers | A sub-theme of the framework focuses on recommendations for support. Providers recommended patients to be provided with devices to facilitate remote monitoring of conditions. This could be combined with in-home testing and a nursing evaluation. Reducing click burden is also important. Patients also recommend supporting by allowing ‘sharing of screens’ in various eHealth types. | | NS | NS | NS | Providing patients with remote monitoring devices and integrating in-home testing and **nursing evaluation**. |
| Kim et al [34] | Older adults (65+), diabetes | Two diabetes clinics | Inform the development of future interventions aimed at promoting mobile health use among older adults and a reference for designers of apps tailored to specific needs. | Social support (from family, friends, and other meaningful relationships) has a direct positive effect on eHealth literacy (B=0·656, p<0·001), and on mHealth use (b=0·218, p=0·014). ‘Social support’ also mediates eHealth literacy (b=0·348, p<0·001). Also, app design aesthetics mediates the relationship between social support and mHealth use (b=0·190, p=0·023). | | NS | NS | NS | Application design aesthetics, such as font size and color, should be considered to develop tailored mobile health apps that mediate social support mediate the relationship between **social support** and use of these applications. |
| Marston et al [37] | Older adults (65+) | Older people forum and charity centre | The Technology in Later Life (TILL) project aimed to examine experiences of older adults with technology. | Survey results did not focus on support. Focus group results showed learning opportunities is a facilitator of technology use. | | NS | NS | NS | **Peer-to-peer** learning and support from peers should be available in **community spaces, such as libraries or cafes**, and online support is recommended. |
| Lee et al [35] | Patients (> 19), diabetes | Outpatient clinics, multiple hospitals | Examine relationships among eHealth literacy, self-efficacy, social support, and self-management in patients with diabetes type 2· | No significant relationship was found from social support to diabetes self-management. There was a significant positive relationship between social support and self-efficacy (b=0·32, p<0·001). | | NS | NS | NS | Combine strategies for self-efficacy and **social support** should achieve synergistic effects when providing eHealth literacy programs to patients with diabetes. |
| Lin et al [36] | Participants (45+) | Four local hospitals | Investigate, amongst others, the relationship among use of library and community activities and eHealth literacy of the target group. | The sample was coded into two groups based on their reported use of library or community services. Participants who used library or community activities reported higher eHealth literacy (1·6+/- 1·90 vs. 0·79 +/- 1·46, p<0·05). | | NS | NS | NS | **Libraries and community activities** contribute to higher eHealth literacy. Librarians can play a key role in providing eHealth services in communities. |
| Pack et al [38] | Patients of Health Center (18+) | Community Health Center Network | Gain insight into eHealth use and needs among patient participants. | One fourth of survey respondents noted portal sigh up was unsuccessful. Of these non-users thirty-eight·8% said sending instructions via text would help and 25·2% wanted a video, 33·4% wanted in-home support. In interviews printing was suggested to read while doing. In-person was preferred to have a live demonstration. Most users also indicated to have received help, for example at the health center. | | NS | NS | NS | Written and in-person support by front-desk staff, clear instructions, and **digital health navigators** in hospitals are essential for users to engage with eHealth tools. |
| Radovanovic et al [39] | Various adults India and Africa | Various: Governmental Initiatives, International Labour Organization | Identify KPIs for sustainable development related to digital literacy | Main findings show: a skills-driven community is equipping youth with needed digital skills, a potential for vocal assistance rather than voice to support the use of ICT, | | NS | NS | NS | A **skills-driven community** is equipping youth with needed digital skills, |
| Shahid et al [40] | Clients, Health care providers, Managers, Organizational Leaders | Primary Care Site, Home Care Site, Community Support Services and Hospital-based mental health services | Outline experiences of health care organizations rapidly implementing eHealth during the COVID-19 pandemic. | In most cases preparation for eHealth involved training sessions for clients the day prior to video visits. When personnel were not available reliance was put on informal support in the clinics. In some cases, staff members went in-person to clients homes to ensure the technology was working. | | Informal supporters | NS? | COVID-19 | **Community engagement** is critical for eHealth education, but sustainability of volunteer support should be investigated for long-term success. |
| Van Middelaar et al [41] | Older adults with risk of cardiovascular diseases (65+) | Primary Care | Assess which factors influence initial and sustained engagement of an interactive internet platform. Assess people’s views on implementation of such a platform. | Stimulation of a known CVD coach helps in using the platform. Trust was important for this. | | Remote support health coach | NS | NS | Tailor eHealth based on individual preferences and readiness for change to increase engagement and platform use. **Stimulation of a known CVD coach** helps in using the platform. Trust was important for this. |
| Williams et al [42] | Individuals (21+), > 2 chronic conditions and their care managers | Commercial Insurance Organization | Describe early implementation challenges and stakeholder-driven process adoptions by comparing high touch (primarily face-to-face) and high tech (via remote care platform) care. Provision of iPhones. | Challenges related to using smartphones and to Internet connections. Three solutions were offered: (1) in-person technology support visits, (2) technology user guides and (3) condition specific text message check-ins. After the in-person visits a 15% increase in video visit completion was noted. Of the 379 participants getting a user guide, 179 completed the care. Care plans were centered around individual care management needs. | | NS | NS | NS | Key stakeholders include **care providers** and implementation teams and **community organizations** that represent the population of interest. |
| Wu et al [43] | Older people (60+), > 1 chronic disease | 4 Community Home-based Aging institutions | Explore potential mechanisms between self-management, social support, eHealth literacy and self-efficacy in people with chronic NCDs | Social support was a direct positive predictor of eHealth literacy (b=0·223, p<0·001). Yet, social support negatively affected self-management (b=-0·034) in this population, although this association is non-significant. | | NS | NS | NS | Excessive social support may cause dependence, weakening self-management. **Community-based aging institutions** should promote eHealth use for NCD management. |
| Zhao et al [44] | Older patients (60+) | Hospital wards, Co-developed Transplant Department | Explore how interactions between older adults and the outside world may shape their eHealth use and how these actions might narrow the age-based digital divide | Three pathways along which informants built digital capabilities through:   1. the social core network: family could hamper the development by too much dependence, but could also foster development by relying on interactions for building capabilities.   *sourcing informationà filtering informationà practicing eHealth with direct supportà abstracting experiences to develop basic capabilities.*   1. expanded social network: close contacts such as friends, colleagues and neighbors.   *sourcing information à assessing information through comparison with life and work experiences à experiment and learn from others in network à create a learning method to develop intermediate capabilities.*   1. wide public network: extended info from family and close relations to public sources   *actively searching info from public sources à cross-checking info multiple sources à building self-confidence in using eHealth à forming common language and shared knowledge to develop advanced capabilities* | | NS | National Strategy ‘Healthy China’ to empower older adults to engage in healthy ageing behavior | NS | Encourage **social interaction** between older adults and their **communities** and promote trial-and-error training to build trust in eHealth. **Governments** should implement policies to create opportunities for older adults to experiment with eHealth. |

**References**

26. Arighi A, Fumagalli GG, Carandini T, et al. Facing the digital divide into a dementia clinic during COVID-19 pandemic: caregiver age matters. Neurol Sci. Apr 2021;42(4):1247-1251. [doi: 10.1007/s10072-020-05009-w] [Medline: 33459891]

27. Chen K, Davoodi NM, Strauss DH, et al. Strategies to ensure continuity of care using telemedicine with older adults during COVID-19: a qualitative study of physicians in primary care and geriatrics. J Appl Gerontol. Nov 2022;41(11):2282-2295. [doi: 10.1177/07334648221109728] [Medline: 35711106]

28. Curran VR, Hollett A, Peddle E. Patient experiences with virtual care during the COVID-19 pandemic: phenomenological focus group study. JMIR Form Res. May 1, 2023;7:e42966. [doi: 10.2196/42966] [Medline: 37036827]

29. Han S, Nam SI. Creating supportive environments and enhancing personal perception to bridge the digital divide among older adults. Educ Gerontol. Aug 3, 2021;47(8):339-352. [doi: 10.1080/03601277.2021.1988448]

30. Hayat TZ, Brainin E, Neter E. With some help from my network: supplementing eHealth literacy with social ties. J Med Internet Res. Mar 30, 2017;19(3):e98. [doi: 10.2196/jmir.6472] [Medline: 28360024]

31. Hodge H, Carson D, Carson D, Newman L, Garrett J. Using internet technologies in rural communities to access services: the views of older people and service providers. J Rural Stud. Aug 2017;54:469-478. [doi: 10.1016/j.jrurstud.2016.06.016]

32. Jokisch MR, Schmidt LI, Doh M. Acceptance of digital health services among older adults: findings on perceived usefulness, self-efficacy, privacy concerns, ICT knowledge, and support seeking. Front Public Health. 2022;10:1073756. [doi: 10.3389/fpubh.2022.1073756] [Medline: 36582385]

33. Khairat S, Chourasia P, Muellers KA, Andreadis K, Lin JJ, Ancker JS. Patient and provider recommendations for improved telemedicine user experience in primary care: a multi-center qualitative study. Telemed Rep. 2023;4(1):21-29. [doi: 10.1089/tmr.2023.0002] [Medline: 36950478]

34. Kim M, Kim B, Park S. Social support, eHealth literacy, and mHealth use in older adults with diabetes: moderated mediating effect of the perceived importance of app design. Comput Inform Nurs. Feb 1, 2024;42(2):136-143. [doi: 10.1097/CIN.0000000000001081] [Medline: 38129323]

35. Lee EH, Lee YW, Kang EH, Kang HJ. Relationship between electronic health literacy and self-management in people with type 2 diabetes using a structural equation modeling approach. J Nurs Res. Jan 1, 2024;32(1):e315. [doi: 10.1097/jnr.0000000000000588] [Medline: 38128065]

36. Lin Z, Zhang Y, Matteson M, et al. Older adults’ eHealth literacy and the role libraries can play. J Lib Inf Sci. Sep 2021;53(3):488-498. [doi: 10.1177/0961000620962847]

37. Marston HR, Genoe R, Freeman S, Kulczycki C, Musselwhite C. Older adults’ perceptions of ICT: main findings from the Technology In Later Life (TILL) study. Healthcare (Basel). Jul 4, 2019;7(3):86. [doi: 10.3390/healthcare7030086] [Medline: 31277387]

38. Pack AP, Rusca P, Llaneza J, et al. Optimizing telehealth services: a mixed-methods needs assessment conducted among community health center patients. Med Care. Jan 1, 2024;62(1):30-36. [doi: 10.1097/MLR.0000000000001932] [Medline: 37796220]

39. Radovanović D, Holst C, Belur SB, et al. Digital literacy key performance indicators for sustainable development. SI. 2020;8(2):151-167. [doi: 10.17645/si.v8i2.2587]

40. Shahid S, Hogeveen S, Sky P, et al. Health equity related challenges and experiences during the rapid implementation of virtual care during COVID-19: a multiple case study. Int J Equity Health. Mar 11, 2023;22(1):44. [doi: 10.1186/s12939-023-01849-y] [Medline: 36906566]

41. van Middelaar T, Beishuizen CRL, Guillemont J, et al. Engaging older people in an internet platform for cardiovascular risk self-management: a qualitative study among Dutch HATICE participants. BMJ Open. Jan 21, 2018;8(1):e019683. [doi: 10.1136/bmjopen-2017-019683] [Medline: 29358447]

42. Williams K, Markwardt S, Kearney SM, et al. Addressing implementation challenges to digital care delivery for adults with multiple chronic conditions: stakeholder feedback in a randomized controlled trial. JMIR Mhealth Uhealth. Feb 1, 2021;9(2):e23498. [doi: 10.2196/23498] [Medline: 33522981]

43. Wu Y, Wen J, Wang X, et al. Chinese community home-based aging institution elders’ self-management of chronic non-communicable diseases and its interrelationships with social support, e-health literacy, and self efficacy: a serial multiple mediation model. Patient Prefer Adherence. 2023;17:1311-1321. [doi: 10.2147/PPA.S412125] [Medline: 37255948]

44. Zhao Y, Zhang T, Dasgupta RK, Xia R. Narrowing the age‐based digital divide: Developing digital capability through social activities. Information Systems Journal. Mar 2023;33(2):268-298. [doi: 10.1111/isj.12400]
