# Supplementary material for: Support Strategies and Interventions for eHealth Inclusion: Scoping Review
Source: J Med Internet Res. 2025 Dec 12;27:e79760. doi: 10.2196/79760 (PMC12700317; doi:10.2196/79760)
Supplement: Multimedia Appendix 7 [file jmir-v27-e79760-s007.docx]

| **Table S4. Results of intervention studies.** | | | | | | | | | | |
| --- | --- | --- | --- | --- | --- | --- | --- | --- | --- | --- |
|  | **Intervention** | **Population** | **Intervention** | | **Outcome** | | **Intervention** |  |  |  |
| **Authors** | **Project** | **Users** | **Organization: Support Site** | **Support actors** | **Intended value** | **Realized value** | **External Stakeholders** | **Legislative Context** | **Temporal Dynamics** | **Future intentions** |
| Alon et al [45] | Digital Outreach for Obtaining Resources and Skills (DOORs) | Adults, with serious mental illness | Community club houses | Educated DOORs instructors after completion digital navigator training | Help mitigate the second digital divide across the globe | Significant improvement (P<0·05) in 8/29 digital skills assessed, 6/29 remained the same | NS | NS | COVID-19 | Expand modules to allow for customized support. |
| Antonio et al [46] | FQHC volunteers | Patients (18+) | Primary Care: Federally Qualified Health Center | FHQC volunteers: graduate student research assistants | Reducing cognitive load and enhancing self-efficacy | During the helping session, eleven participants attempted to sign up for the patient portal of which five were successful. Helpers resolved issues during all 16-video visit test runs by supporting in device settings, browsers and external programs closing. | NS | NS | COVID-19 | Future studies should design blended strategies combining both human support and improved access to technologies |
| Bevilacqua et al [47] | ACCESS | Older adults | Online Platform: GoToMeeting | Trained facilitators via online platform, and students via inter-generational learning | Inform and educate on eHealth, teach eHealth skills and support behavior change | After the course, the eHealth literacy score (eHEALS) of older adults overall had a significant negative correlation relationship with positive skills of using technology (SOTU) (p = 0·048 and p = 0·032). | NGO: to recruit participants | NS | COVID-19 | Suggestion for future evidence-based interventions focusing also on the hard-to-reach people. |
| Camacho et al [48] | Digital Outreach for Obtaining Resources and Skills (DOORs) | Clubhouse participants and IPU participants | Clubhouses, inpatient psychiatric unit (IPU) | Trained digital navigators | Teaching participants skills via the use of smartphones. | Clubhouse participants initially displayed deficiencies on 24 of 29 functional skills. After offering the curriculum 25 of 29 skills were sufficient.  IPU participants showed no deficiencies in functional skills pre-intervention, and although skills improved, no significant differences were observed between pre- and post-intervention. | NS | NS | NS | Using the basic skills of the DOORs curriculum as a starting course/key to DOORs course. |
| Chu et al [49] | Video Visits for Elderly Patients (VVEP) team | Older adults (65+) | General Internal Medicine Primary Care Practice | Clinical research coordinators, medical students, GIM physicians | Direct supportive assistance and connection to community-based services | The support team was able to successfully video-enable devices for 149 (77·six%) of the older adults participating. Of patients reached by the VVEP team, 40% completed their primary care visit over video, 26·5% completed a tele- phone visit, 29·6% canceled their scheduled visit, 2·1% no- showed, and 1·4% had an in-person visit. | NS | NS | COVID-19: forced telephone support | In-person support, or using the developed telephone tip sheet |
| Drazich et al [50] | ADD2PCOR, | Community stakeholders | Community Organizations: Online | Instructions by research team | A time-efficient, cost-effective and feasible training course to bridge the digital divide | Most participants were extremely satisfied with the program (11/16, 69%) and strongly agreed that the experience was worthwhile (15/16, 94%). A comparison of the combined total subdomains scores before and after the program showed an increase in score by thirteen·91 (scale 0-48) for the frequency of technology use. | Partnership community patient recruitment | NS | COVID-19: forced virtual support | For future studies to implement a similar program in a larger sample. |
| Gusdorf et al [51] | Vanderbilt Telehealth Volunteer Program (VTVP) | Patients, in general | University Medical Center | Medical students | Evaluate the effect of an intervention using pre-visit telephone calls | Patients who received a call from a medical student had decreased rates of failed video visits (OR 0·fifty-four; 95% CI: 0·48–0·60). | NS | NS | COVID-19 pandemic | Evaluating this at a multi-center or national level. |
| Hawley et al [52] | In-home team member Veterans Affairs (VA) | Older adults, veterans (65+) >2 chronic medical conditions, no dementia | Veterans Affairs Facility | VA employee, Study member, In-home team member, Pharmacist | Gain insight into facilitators and barriers to video visits for older adults to offer evidence-based solutions to clinicians to address these gaps | 10 (50% of the) participants required assistance before or during the visit. Seven required in-home assistance beforehand, six required assistances during. The pharmacist could solve challenges for three of them, for the other 3 the in-home team member was required to help. | NS | NS | COVID-19 limited visits | Distinction between low- and high-effort (LE and HE) solutions to implement to deal with technology challenges, since in-team member seems not always feasible. Solutions: (1) identify and prepare champions (LE: pre-video visit online help; HE: offer focus on training and in-home help), (2) involve patients and family member (LE: inquire about tech-savvy family and schedule practice sessions with them; HE: schedule in-person training with family days before and when family is available), (3) Assess readiness (LE: troubleshooting guides, inquiry on suitableness devices; HE: in-person test visit and on-demand help access during all video visits), (4) Promote adaptability (LE: set protocol for clinicians on what to do with tech challenges; HE: evaluate all video visits), (5) Create a collaborative learning environment (LE: Share best practices within local site; HE: Share best practices outside local site) |
| Hernandez-Ramos et al [53] | One-on-one staff-patient partnerships DIAMANTE diabetes app (DIAMANTE) | Patients, limited digital literacy | Public health care system | Staff | Develop practical guidelines for inter-based remote recruitment of populations having access to technology, but facing barriers for use | 8 of 11 (73%) patients in the COVID-19 cohort had never participated in a remote visit for research in the past, but all 11 (100%) patients stated that they enjoyed participating in remote visits for research. | NS | NS | COVID-19: transition to development remote study | Guidelines to use for increased access to technology |
| Hoffman et al [54] | Inter-organizational Telehealth Program | Patients, in general | Hub- and spoke Academic Medical Center: composed of five community hospitals. | MyChart Support Team and IT personnel | Sustaining and growing telehealth | Not specified. | NS | Connecticut state telehealth law requirements, CSME and AGGE guidelines, HIPAA | COVID-19: redefined roles | Expanded patient support, Ongoing internal research into healthcare and technological disparities. Patient-facing IT support phone number. Tiered support for patients over sixty-five, then under 18, then others. |
| Jones et al [55] | Plymouth SeniorNet (PSN volunteers) | Older adults (65+) | At home via local advertisements | Volunteers (50+) | Support volunteers over fifty to help people over 65 go online. | N = 144 beneficiaries supported people: a) one-on-one at home for n = 58 b) in small group sessions by n = 86· Data was available on n = 110· Use of the Internet for health was least valued compared to other values. | Funder: UK’s Big Lottery | NS | NS | Combination of one-on-one and group support. |
| Lee and Kim [56], 2019 | Intergenerational Mentor-Up (IMU) | Older adults (65+) | Recruitment via two senior centers and 2 senior housing facilities. Intervention at a Public University. | Instructed youth mentions undergraduate student as part of a course assignment | Intergenerational exchanges via youth-led tutorials on health information technologies. | Regarding eHealth literacy the older adults showed significant improvement at posttest (t = -5·89, p< .001, d = -0·79). Willingness to use also significantly increased at post-test (t = -7·99, p < .001, d = -1·08). Qualitative data shows individualized training, and intergenerational interactions can decrease their anxiety and boost their confidence. | NS | NS | NS | A need for more and longer sessions is stressed by the users. |
| Lim et al [57] | Project Wire Up | Vulnerable older adults with low SES | NPO TriGen LtD and General Hospital | Volunteers Wire Up and Staff Grassroot Organization | Improving digital access and literacy levels to address social isolation and lack of access to essential services | Grassroot organizations played a part in ensuring the success of the digital access and literacy program. Older adults would be prepared and assisted via these organizations via door-to-door visits. It was helpful to approach people in their mother tongue. Also, pacing the teaching and repeating sessions showed greater results, as well as increasing the font size, contrast and brightness of a device. | Telecommunication companies and Statuary Board Authority to equip older adults with the tools to facilitate digital literacy | Singapore law: individuals not allowed to purchase telephone connections for others | COVID-19: started when in-person visits were allowed | Need for public and private efforts to provide high-quality mobile devices |
| Lyles et al [58] | MYSFHEALTH platform training (MYSFHEALTH) | Patients, in general | General Hospital and two advisory boards | Trained research assistants | Increased portal use among diverse patients | During the follow-up period, eighteen participants (21%) logged in to the portal, and an extra 17 (20%) signed up for a clinic visit. Those who watched more than one video had a higher login rate (23% vs. 15%), yet not statistically significant. eHealth literacy scores increased significantly (14·4 to 16·2, p<0·001), but interest in using the portal decreased from 53% to 39% (p=0·01). The training mode (in-person vs. take-home) did not affect portal use. | NS | NS | NS | This training was not successful in getting most patients to become portal users. |
| Maliwichi et al [59] | Infomediaries, | Citizens, in general | Use Case: Chipatala cha pa Foni (CCPF), Malawi’s Health and Nutrition Hotline | Infomediaries (e.g., community volunteers) | Using volunteers within the community for amongst others mobile phone access | The likelihood of the availability of technologies and grid power has the potential to exacerbate inequalities in poor resource settings. | NS | NS | COVID-19: travel restrictions interviews | Further identifying and using intermediaries |
| Mechanic et al [60] | Patient Navigator Program (PNP) | Patients, primary and gerontology clinic | Academic Hospital: Primary and Gerontology Clinics | Trained telehealth Patient Navigator | Reduce barriers to successful video visit use | Overall, patients receiving the intervention had higher odds of attending video visit (OR 2·zero; 95% CI, 1·6-2·6). For both clinics, the intervention group had a 21% absolute increase in video visit success rate compared with the comparator group (81% vs 60%; P < .001). Missed appointment and cancellation rates were 8% lower in intervention group. | NS | NS | NS | Cost-effectiveness analysis suggesting implementing a telehealth patient navigator may be of high value for healthcare systems. |
| Ramsey et al [61] | MyChart Genius | Patients > 13 years old, and in the case of youth: their proxy users | Primary Care Clinic | MyChart Geniuses: Volunteer staff of premedical and medical students | Determine the feasibility, acceptability, and preliminary effectiveness of assistance of MyChart Geniuses | Increased MyChart sign-ups from 478/995 (48%) to 814/1376 (59%). The Geniuses signed up n = 84 patients, of which 17 (28%) activated their accounts by the end of the project. | NS | Proxy access only allowed for patients <13 years old. | pre-COVID | Portal usage following sign-ups in terms of activation could be further explored. |
| Rodriguez et al [62] | Digital Health Navigators (DHN) | Patients, diabetes | Primary Care Clinic | Digital Health Navigators | Describe the implementation process of the DHN program and provide a quantitative analysis of the program | Able to enroll 121 of the reached patients (30·nine%) | NS | 21^st^ Cures Act: shift to telemedicine, increasing patient access to their data. | COVID-19: review portal use data | Give recommendations for others, such as: align the DHN program with organizational goals to ensure support for programs |
| Senteio et al [63] | Intergenerational Technology Transfer (ITT) | Older adults (55=), diabetes | Faith Based Organization (FBO) | Younger adults and leaders FBO | Promote inter-generational technology transfer between older adults with diabetes and younger adults connected to them. | Participants felt significantly  more confident in using technology designed to help with  their health (p=0·0002), obtaining help needed to use technol-  ogy (p=0·0003), downloading a health application (p=0·0005),  helping others use technology (p=0·0003), and felt better  when others help them use technology (p=0·0039). They did not feel significantly more confident in using websites to get health information (p=0·1534). | Community centers and FBOs for participant recruitment | NS | NS | For practitioners to identify a support system to help chronic disease patients in using technology. |
| Taylor et al [64] | Initial Telehealth Education Modules (i-TEMs) | Interviews: Older adults (60+)  Survey: 60+ and > 1 chronic condition | Recruitment of community volunteers via the University Center for Excellence in Aging for interviews. | Web-based modules (https://accesstelehealthtraining.org/telehealth-training/) | Evaluate the extent to which TEMs improve self-perceived telehealth competency for older adults | Telehealth competency increased significantly for 12/13 items (p<0·05). | Simulation center for practice examples | NS | COVID-19: use of eHealth increased | Create training reflecting real practice, and involve the target group |
| Worster et al [65] | Telehealth Task Force (TTF), | Patients, cancer | Cancer Center | Nurse, IT, care coordinators | Assess the impact of the TTF on improving the uptake of telehealth | Of the scheduled appointment in the cancer center 0·52% (n = 79) were telehealth pre-intervention, 25·57% (n = 4287) during intervention and 19·85% (n = 3446) post. | NS | HIPAA and billing regulations | COVID-19: stay at home orders | A broader picture of challenges faced and opportunities for collaborations around interventions to address digital literacy |

**References**

45. Alon N, Perret S, Torous J. Working towards a ready to implement digital literacy program. Mhealth. 2023;9:32. [doi: 10.21037/mhealth-23-13] [Medline: 38023777]

46. Antonio MG, Williamson A, Kameswaran V, et al. Targeting patients’ cognitive load for telehealth video visits through student-delivered helping sessions at a United States federally qualified health center: equity-focused, mixed methods pilot intervention study. J Med Internet Res. Feb 1, 2023;25:e42586. [doi: 10.2196/42586] [Medline: 36525332]

47. Bevilacqua R, Strano S, Di Rosa M, et al. eHealth literacy: from theory to clinical application for digital health improvement. Results from the ACCESS training experience. Int J Environ Res Public Health. Nov 10, 2021;18(22):11800. [doi: 10.3390/ijerph182211800] [Medline: 34831555]

48. Camacho E, Torous J. Impact of digital literacy training on outcomes for people with serious mental illness in community and inpatient settings. Psychiatr Serv. May 1, 2023;74(5):534-538. [doi: 10.1176/appi.ps.20220205] [Medline: 36164771]

49. Chu JN, Kaplan C, Lee JS, Livaudais-Toman J, Karliner L. Increasing telehealth access to care for older adults during the COVID-19 pandemic at an academic medical center: Video Visits for Elders Project (VVEP). Jt Comm J Qual Patient Saf. Mar 2022;48(3):173-179. [doi: 10.1016/j.jcjq.2021.11.006] [Medline: 35027304]

50. Drazich BF, Nyikadzino Y, Gleason KT. A program to improve digital access and literacy among community stakeholders: cohort study. JMIR Form Res. Nov 10, 2021;5(11):e30605. [doi: 10.2196/30605] [Medline: 34757316]

51. Gusdorf RE, Shah KP, Triana AJ, et al. A patient education intervention improved rates of successful video visits during rapid implementation of telehealth. J Telemed Telecare. Sep 2023;29(8):607-612. [doi: 10.1177/1357633X211008786] [Medline: 33975506]

52. Hawley CE, Wagner C, Venegas MD, et al. Connecting the disconnected: leveraging an in‐home team member for video visits for older adults. J Am Geriatr Soc. May 2024;72(5):1408-1419. URL: <https://agsjournals.onlinelibrary.wiley.com/toc/15325415/72/5> [doi: 10.1111/jgs.18663] [Medline: 37960887]

53. Hernandez-Ramos R, Aguilera A, Garcia F, et al. Conducting internet-based visits for onboarding populations with limited digital literacy to an mHealth intervention: development of a patient-centered approach. JMIR Form Res. Apr 29, 2021;5(4):e25299. [doi: 10.2196/25299] [Medline: 33872184]

54. Hoffman PE, London YR, Weerakoon TS, DeLucia NL. Rapidly scaling video visits during COVID-19: The ethos of virtual care at Yale Medicine. Healthcare (Basel). Dec 2020;8(4):100482. [doi: 10.1016/j.hjdsi.2020.100482]

55. Jones RB, Ashurst EJ, Atkey J, Duffy B. Older people going online: its value and before-after evaluation of volunteer support. J Med Internet Res. May 18, 2015;17(5):e122. [doi: 10.2196/jmir.3943] [Medline: 25986724]

56. Lee OEK, Kim DH. Bridging the digital divide for older adults via intergenerational mentor-up. Res Soc Work Pract. Oct 2019;29(7):786-795. [doi: 10.1177/1049731518810798]

57. Lim HA, Lee JSW, Lim MH, et al. Bridging connectivity issues in digital access and literacy: reflections on empowering vulnerable older adults in Singapore. JMIR Aging. May 3, 2022;5(2):e34764. [doi: 10.2196/34764] [Medline: 35503520]

58. Lyles CR, Tieu L, Sarkar U, et al. A randomized trial to train vulnerable primary care patients to use a patient portal. J Am Board Fam Med. 2019;32(2):248-258. [doi: 10.3122/jabfm.2019.02.180263] [Medline: 30850461]

59. I Maliwichi P, Chigona W. Towards a framework on the use of infomediaries in maternal mHealth in rural Malawi. IJIKM. 2022;17:387-411. [doi: 10.28945/5015]

60. Mechanic OJ, Lee EM, Sheehan HM, et al. Evaluation of telehealth visit attendance after implementation of a patient navigator program. JAMA Netw Open. Dec 1, 2022;5(12):e2245615. [doi: 10.1001/jamanetworkopen.2022.45615] [Medline: 36480202]

61. Ramsey A, Lanzo E, Huston-Paterson H, Tomaszewski K, Trent M. Increasing patient portal usage: preliminary outcomes from the MyChart Genius Project. J Adolesc Health. Jan 2018;62(1):29-35. [doi: 10.1016/j.jadohealth.2017.08.029] [Medline: 29169768]

62. Rodriguez JA, Charles JP, Bates DW, Lyles C, Southworth B, Samal L. Digital healthcare equity in primary care: implementing an integrated digital health navigator. J Am Med Inform Assoc. Apr 19, 2023;30(5):965-970. [doi: 10.1093/jamia/ocad015] [Medline: 36795062]

63. Senteio CR, Hershey DS, Campbell T, Mandal S. Intergenerational technology transfer: enhancing African American older adults’ self-efficacy for diabetes self-management. Prog Community Health Partnersh. 2021;15(4):e5. [doi: 10.1353/cpr.2021.0048] [Medline: 34975018]

64. Taylor S, Souza S, Little L, Odiaga J. Enhancing telehealth competency: development and evaluation of education modules for older adults. OTJR (Thorofare N J). Jul 2023;43(3):478-486. [doi: 10.1177/15394492231153115] [Medline: 36757088]

65. Worster B, Waldman L, Garber G, et al. Increasing equitable access to telehealth oncology care in the COVID-19 national emergency: creation of a telehealth task force. Cancer Med. Feb 2023;12(3):2842-2849. [doi: 10.1002/cam4.5176] [Medline: 36210751]
