# Supplementary material for: Support Strategies and Interventions for eHealth Inclusion: Scoping Review
Source: J Med Internet Res. 2025 Dec 12;27:e79760. doi: 10.2196/79760 (PMC12700317; doi:10.2196/79760)
Supplement: Checklist 1 [file jmir-v27-e79760-s008.pdf]

Preferred Reporting Items for Systematic reviews and Meta-Analyses extension for Scoping Reviews (PRISMA-ScR) Checklist

SECTION	ITEM	PRISMA-ScR CHECKLIST ITEM	REPORTED ON PAGE #
**TITLE**			
Title	1	Identify the report as a scoping review.	✓ p.1<br>Title*: Support Strategies and Interventions for eHealth Inclusion: A Scoping Review.*
**ABSTRACT**			
Structured summary	2	Provide a structured summary that includes (as applicable): background, objectives, eligibility criteria, sources of evidence, charting methods, results, and conclusions that relate to the review questions and objectives.	p.1<br>A structured summary was provided. However, not all eligibility criteria are mentioned in the abstract.
**INTRODUCTION**			
Rationale	3	Describe the rationale for the review in the context of what is already known. Explain why the review questions/objectives lend themselves to a scoping review approach.	✓ p.2<br>The rationale is that support interventions to promote eHealth inclusion are fragmented and under-researched, especially regarding effectiveness and collaboration. A scoping review is appropriate to map the breadth of existing strategies and interventions without critical appraisal. See p.2: **"Various fragmented support interventions… Yet, there is a lack of research into their effectiveness and the role of collaboration with other support actors...".**
Objectives	4	Provide an explicit statement of the questions and objectives being addressed with reference to their key elements (e.g., population or participants, concepts, and context) or other relevant key elements used to conceptualize the review questions and/or objectives.	✓ p.2-3<br>The research questions and aim are specified on page 2. Section 2.1 (p.3) explains the PIO elements: empirical studies targeting (potential) patients (P) receiving help or support (I), with outcomes related to eHealth inclusion (O).
**METHODS**			
Protocol and registration	5	Indicate whether a review protocol exists; state if and where it can be accessed (e.g., a Web address); and if available, provide registration information, including the registration number.	✓ p.2<br>The review was pre-registered in OSF: https://doi.org/10.17605/OSF.IO/6QMCJ.
Eligibility criteria	6	Specify characteristics of the sources of evidence used as eligibility criteria (e.g., years considered, language, and publication status), and provide a rationale.	✓ p.3<br>Eligibility criteria are defined in Section 2.1 using the PIO framework. Inclusion: empirical studies (since 2014, English, peer-reviewed) involving (potential) patients (P), receiving help/support (I), and reporting on eHealth inclusion (O).
Information sources*	7	Describe all information sources in the search (e.g., databases with dates of coverage and contact with authors to identify additional sources), as well as the date the most recent search was executed.	✓ p.3<br>A total of 6,425 records were retrieved from PubMed, Scopus, Web of Science, and Embase on January 29, 2024.
Search	8	Present the full electronic search strategy for at least 1 database, including any limits used, such that it could be repeated.	✓ Appendix 1/A<br>Full search strategies for all databases are included in Appendix 1/A.
Selection of sources of evidence	9	State the process for selecting sources of evidence (i.e., screening and eligibility) included in the scoping review.	✓ p.3<br>Section 2.3 describes the screening and selection process.

St. Michael's
Inspired Care.
Inspiring Science.

SECTION	ITEM	PRISMA-ScR CHECKLIST ITEM	REPORTED ON PAGE #
Data charting process‡	10	Describe the methods of charting data from the included sources of evidence (e.g., calibrated forms or forms that have been tested by the team before their use, and whether data charting was done independently or in duplicate) and any processes for obtaining and confirming data from investigators.	✓ Appendix 1/A + Section 2.4<br>Section 2.4 explains data extraction using a framework combining PIO and NASSS.
Data items	11	List and define all variables for which data were sought and any assumptions and simplifications made.	✓ Table 1<br>Table 1 provides an overview of extracted data items.
Critical appraisal of individual sources of evidence§	12	If done, provide a rationale for conducting a critical appraisal of included sources of evidence; describe the methods used and how this information was used in any data synthesis (if appropriate).	p.2<br>No critical appraisal was conducted, as the aim was to map all existing evidence regardless of quality.<br>**"Since we aimed to map all existing evidence on eHealth support regardless of methodological quality and risk of bias, critical appraisal – an optional component of the PRISMA-ScR 18 – was not conducted."**
Synthesis of results	13	Describe the methods of handling and summarizing the data that were charted.	Charted data were summarized thematically using the PIO and NASSS frameworks for both strategy and intervention studies.
**RESULTS**			
Selection of sources of evidence	14	Give numbers of sources of evidence screened, assessed for eligibility, and included in the review, with reasons for exclusions at each stage, ideally using a flow diagram.	✓ Figure 1<br>Figure 1 shows the PRISMA flow diagram with screening and exclusion numbers.
Characteristics of sources of evidence	15	For each source of evidence, present characteristics for which data were charted and provide the citations.	✓ Table 4 / Sections 3.1 and 3.4<br>Table 4 lists descriptive characteristics. Section 3.1 describes strategy studies; 3.4 focuses on intervention studies.
Critical appraisal within sources of evidence	16	If done, present data on critical appraisal of included sources of evidence (see item 12).	p.2<br>No critical appraisal was conducted.
Results of individual sources of evidence	17	For each included source of evidence, present the relevant data that were charted that relate to the review questions and objectives.	✓ Table 5 and 6<br>Tables 5 and 6 present data on support actors and outcome measures.
Synthesis of results	18	Summarize and/or present the charting results as they relate to the review questions and objectives.	✓ Section 3.3 and 3.5<br>These sections thematically summarize results of the included studies.
**DISCUSSION**			
Summary of evidence	19	Summarize the main results (including an overview of concepts, themes, and types of evidence available), link to the review questions and objectives, and consider the relevance to key groups.	✓ Section 3.3 & 3.5<br>These sections summarize the main results.
Limitations	20	Discuss the limitations of the scoping review process.	✓ Section 4<br>Limitations are addressed in Section 4.
Conclusions	21	Provide a general interpretation of the results with respect to the review questions and objectives, as well as potential implications and/or next steps.	✓ Section 4<br>✓ Section 4 includes interpretation and potential implications for future research and practice.

SECTION	ITEM	PRISMA-ScR CHECKLIST ITEM	REPORTED ON PAGE #
**FUNDING**			
Funding	22	Describe sources of funding for the included sources of evidence, as well as sources of funding for the scoping review. Describe the role of the funders of the scoping review.	NA

JBI = Joanna Briggs Institute; PRISMA-ScR = Preferred Reporting Items for Systematic reviews and Meta-Analyses extension for Scoping Reviews.
* Where *sources of evidence* (see second footnote) are compiled from, such as bibliographic databases, social media platforms, and Web sites.
† A more inclusive/heterogeneous term used to account for the different types of evidence or data sources (e.g., quantitative and/or qualitative research, expert opinion, and policy documents) that may be eligible in a scoping review as opposed to only studies. This is not to be confused with *information sources* (see first footnote).
‡ The frameworks by Arksey and O'Malley (6) and Levac and colleagues (7) and the JBI guidance (4, 5) refer to the process of data extraction in a scoping review as data charting.
§ The process of systematically examining research evidence to assess its validity, results, and relevance before using it to inform a decision. This term is used for items 12 and 19 instead of "risk of bias" (which is more applicable to systematic reviews of interventions) to include and acknowledge the various sources of evidence that may be used in a scoping review (e.g., quantitative and/or qualitative research, expert opinion, and policy document).

From: Tricco AC, Lillie E, Zarin W, O'Brien KK, Colquhoun H, Levac D, et al. PRISMA Extension for Scoping Reviews (PRISMAScR): Checklist and Explanation. Ann Intern Med. 2018;169:467–473. doi: 10.7326/M18-0850.
